# Pan-cancer analysis of necroptosis-related gene signature for the identification of prognosis and immune significance

**DOI:** 10.1007/s12672-022-00477-2

**Published:** 2022-03-21

**Authors:** Jincheng Ma, Yan Jin, Baocheng Gong, Long Li, Qiang Zhao

**Affiliations:** 1 Tianjin Key Laboratory of Cancer Prevention and Therapy, Department of Pediatric Oncology, Tianjin Medical University Cancer Institute and Hospital, National Clinical Research Center for Cancer, Tianjin’s Clinical Research Center for Cancer, Tianjin, China; 2 Key Laboratory of Immune Microenvironment and Diseases of Educational Ministry of China, Department of Immunology, The Province and Ministry Co-sponsored Collaborative Innovation Center for Medical Epigenetics, Tianjin Medical University, Tianjin, China

**Keywords:** Cancer, Necroptosis, Prognosis, Risk score, Tumor immune infiltration

## Abstract

**Background:**

Necroptosis is a novel programmed cell death mode independent on caspase. A number of studies have revealed that the induction of necroptosis could act as an alternative therapeutic strategy for drug-resistant tumors as well as affect tumor immune microenvironment.

**Methods:**

Gene expression profiles and clinical data were downloaded from XENA-UCSC (including The Cancer Genome Atlas and Genotype-Tissue Expression), Gene Expression Omnibus, International Cancer Genome Consortium and Chinese Glioma Genome Atlas. We used non-negative matrix factorization method to conduct tumor classification. The least absolute shrinkage and selection operator regression was applied to establish risk models, whose prognostic effectiveness was examined in both training and testing sets with Kaplan–Meier analysis, time-dependent receiver operating characteristic curves as well as uni- and multi-variate survival analysis. Principal Component Analysis, t-distributed Stochastic Neighbor Embedding and Uniform Manifold Approximation and Projection were conducted to check the risk group distribution. Gene Set Enrichment Analyses, immune infiltration analysis based on CIBERSORT, EPIC, MCPcounter, ssGSEA and ESTIMATE, gene mutation and drug sensitivity between the risk groups were also taken into consideration.

**Results:**

There were eight types of cancer with at least ten differentially expressed necroptosis-related genes which could influence patients’ prognosis, namely, adrenocortical carcinoma (ACC), cervical squamous cell carcinoma and endocervical adenocarcinoma (CESC), acute myeloid leukemia (LAML), brain lower grade glioma (LGG), pancreatic adenocarcinoma (PAAD), liver hepatocellular carcinoma (LIHC), skin cutaneous melanoma (SKCM) and thymoma (THYM). Patients could be divided into different clusters with distinct overall survival in all cancers above except for LIHC. The risk models could efficiently predict prognosis of ACC, LAML, LGG, LIHC, SKCM and THYM patients. LGG patients from high-risk group had a higher infiltration level of M2 macrophages and cancer-associated fibroblasts. There were more CD8+ T cells, Th1 cells and M1 macrophages in low-risk SKCM patients’ tumor microenvironment. Gene mutation status and drug sensitivity are also different between low- and high-risk groups in the six cancers.

**Conclusions:**

Necroptosis-related genes can predict clinical outcomes of ACC, LAML, LGG, LIHC, SKCM and THYM patients and help to distinguish immune infiltration status for LGG and SKCM.

**Supplementary Information:**

The online version contains supplementary material available at 10.1007/s12672-022-00477-2.

## Introduction

Although recent authoritative statistics showed that the death rate of cancer declined over the past 30 years, cancer remains one of the primary causes of death worldwide no matter in developed or developing countries, which greatly increases economic burden and seriously affects life quality [1]. The occurrence and development of tumor involves a series of extremely complex biological processes, and the treatment effect of many tumors is still not satisfactory even under the combination of multiple therapies. It is urgent and of great importance to find novel insights and effective agents for cancer.

The resistance to cell death has been identified as one of the most important characters of malignant tumors [2]. Classical theory divided cell death forms into apoptosis and necrosis, according to the whether it’s under the programmed regulation of genetic materials [3]. However, in the 1990s, a new pattern of necrosis-like cell death featured by non-caspase dependency gradually emerged. Researchers found that, under the inhibition of key proteins in apoptosis pathway [such as Caspase-8 or Fas-associated protein with death domain (FADD)] and the stimulation of tumor necrosis factor α (TNF-α), the cell morphology was similar to the necrotic cell [4, 5]. Then, at the beginning of the twenty-first century, the concept and process of programmed necrosis or necroptosis was gradually proposed and elaborated [6–8]. In 2018, the Nomenclature Committee on Cell Death (NCCD) officially defined this special form of cell death as necroptosis [9]. Unlike apoptosis which involves kinds of morphological changes, such as cell shrinkage and detachment from the surrounding cells, nucleoplasm concentration, fragmentation of nuclear membrane and nucleolus as well as the appearance of apoptotic bodies, several special biological events occur in cells undergoing necroptosis, including the damage of membranes, disorder of metabolism and the extravasation of inflammatory substances [8]. Necroptosis and apoptosis share the same initiating stage. When tumor necrosis factor receptor 1 (TNFR1) on the cell membrane surface is activated by TNF-α, TNF receptor 1-associated death domain protein (TRADD) and tumor necrosis factor and receptor related factor 2 (TRAF2) will be recruited by its death domain at C-terminal. Subsequently, TRADD and TRAF2 separately recognizes and binds to receptor-interacting protein kinase 1 (RIPK1) and cellular inhibitors of apoptosis 1 and 2 (CIAP1/2), and protein complex scaffold is formed by linear ubiquitin Chain assembly complex (LUBAC). Then, with the combination of these molecules and transforming growth factor-beta (TGF-β) activated kinase 1/TGF-β activated kinase 1 binding protein (TAK1/TAB) complex, the supramolecular structure (TNFR1 Complex I) come into being [10]. The deubiquitination of RIPK1 by the cylindromatosis (CYLD) can result in the cleavage of Complex I and the dissociation of RIPK1 as well as TRADD, where different endings of the cell happen. Complex IIa constituted of TRADD, FADD as well as Caspase-8 and Complex IIb composed of PIPK1, receptor-interacting protein kinase 3 (RIPK3), FADD and Caspase-8 would lead cell to apoptosis. The catalytic activity inhibition of caspase-8 would allow RIPK1 to phosphorylate RIPK3, which recruits mixed lineage kinase domain-like (MLKL) to form necroptosome [11, 12]. MLKL migrates to cell membrane to result in necroptosis.

Necroptosis played an indispensable role in the maintenance of internal environment homeostasis and the progression of several inflammation-related diseases, such as neurodegenerative disease, ischemia–reperfusion injury and pathogen infection [10, 13]. A number of studies have also revealed the significance of necroptosis induction at cancer treatment in recent years, which especially worked for the apoptosis-resistant tumors [14]. Meanwhile, with the rise of immunotherapy, the relationship between different forms of cell death and tumor immunity has gradually attracted extensive attention [15]. There was no effective anti-tumor immune response observed in the tumor area where apoptosis or necrosis occurred. However, increasing number of studies have revealed the influence of necroptosis on tumor immune microenvironment, where the results were opposite in different tumor models. Damage associated molecular patterns (DAMPs) and various cytokines and chemokines which leaked out of necroptotic cells of colon carcinoma and melanoma could strengthen cytotoxic function of CD8+ T cells and the activity of antigen-presenting cells [16–18]. However, the necroptotic cells of pancreatic ductal adenocarcinoma enhanced the immunosuppressive function of tumor-associated macrophage (TAM) by C-X-C motif chemokine ligand 1 (CXCL1) and Mincle signaling [19]. The studies also showed that the synergistic effect of necroptosis-promoting agents and immune checkpoint inhibitors (ICIs) could trigger long-term tumor-suppression effect in mouse models [17, 18], indicating that the necroptosis induction of tumor cell was probably an effective complement to immunotherapy.

In this study, we comprehensively analyzed the necroptosis-related genes in different kinds of cancers based on data from The Cancer Genome Atlas (TCGA), Genotype-Tissue Expression (GTEx), Gene Express Omnibus (GEO), International Cancer Genome Consortium (ICGC) and Chinese Glioma Genome Atlas (CGGA). We developed novel tumor classification and constructed risk models based on necroptosis-related genes to predict patients’ clinical outcomes. Immune infiltration, gene mutation and drug sensitivity were also taken into consideration.

## Methods

### Gene expression and clinical data collection

We obtained gene profiles, clinical features and survival information of 33 TCGA cancers from XENA-UCSC (https://xena.ucsc.edu/). For thirteen types of cancer with no or very limited number of corresponding normal tissue samples (< 10), we obtained gene expression data of normal samples from GTEx at XENA-UCSC, namely, adrenocortical carcinoma (ACC), cervical squamous cell carcinoma and endocervical adenocarcinoma (CESC), lymphoid neoplasm diffuse large B-cell lymphoma (DLBC), glioblastoma multiforme (GBM), acute myeloid leukemia (LAML), brain lower grade glioma (LGG), ovarian serous cystadenocarcinoma (OV), pancreatic adenocarcinoma (PAAD), rectum adenocarcinoma (READ), skin cutaneous melanoma (SKCM), testicular germ cell tumors (TGCT), thymoma (THYM) and uterine carcinosarcoma (UCS). Because of no relevant samples for pheochromocytoma and paraganglioma (PCPG) and sarcoma (SARC) found in GTEx, we only used TCGA data for the analysis. Mesothelioma (MESO) and uveal melanoma (UVM) were excluded from this study, for there were no normal samples in neither TCGA nor GTEx. Necroptosis-related gene list (hsa04217) was found in Kyoto Encyclopedia of Genes and Genomes (KEGG). The details of necroptosis-related genes were shown in Supplementary file 1.

The other cohorts with patients’ clinical and survival information were obtained for ACC, CESC, LAML, LGG, liver hepatocellular carcinoma (LIHC), PAAD, SKCM from GEO, ICGC and CGGA. The details are as listed:

- ACC: GSE19750 [20] https://www.ncbi.nlm.nih.gov/geo/query/acc.cgi?acc=GSE19750.
- GSE33371 [21] https://www.ncbi.nlm.nih.gov/geo/query/acc.cgi?acc=GSE33371.
- CESC: GSE44001 [22] https://www.ncbi.nlm.nih.gov/geo/query/acc.cgi?acc=GSE44001.
- LAML: GSE37642 [23] https://www.ncbi.nlm.nih.gov/geo/query/acc.cgi?acc=GSE37642.
- LGG: CGGA_693, CGGA_325 [24] http://www.cgga.org.cn/.
- LIHC: ICGC (LIRI-JP) https://icgc.org/.
- PAAD: ICGC (PACA-AU) https://icgc.org/.
- SKCM: GSE65904 [25] https://www.ncbi.nlm.nih.gov/geo/query/acc.cgi?acc=GSE65904.

### Identification of differentially expressed necroptosis-related genes (DENGs), survival analysis and tumor classification

To identify DENGs between tumors and the corresponding normal tissues, the “limma” R package was applied, with |log2 (fold change)| > 1 and false discovery rate (FDR) < 0.05 as the thresholds. Then, we conducted survival analysis of DENGs in each particular type of cancer. The cancer types with at least 10 DENGs that significantly influence patients’ overall survival (OS) were selected. Next, we constructed chord diagrams of the prognostic DENGs in the chosen cancers by using “circlize” and “corrplot” R packages, where Pearson correlation analysis was performed. The correlation at protein level was visualized by STRING (Version: 11.5, https://cn.string-db.org/) through “Multiple protein” module with the “Homo sapiens” and “low confidence (0.150)” as the main parameters. Finally, based on prognostic DENGs, we used the non-negative matrix factorization (NMF) to conducted cancer classification. “NMF” R package was used, with “brunet”, “10 iterations” and “clusters k ranks from 2 to 10” as the main parameters. Kaplan–Meier analysis was performed between patients’ survival and the different clusters, where four survival endpoints were taken into consideration, namely, OS, disease specific survival (DSS), progression free survival (PFS) and disease free survival (DFS).

### Construction and validation of DENGs-based risk model

First, batch corrections were performed between TCGA cohorts and the corresponding additional cohorts of the selected cancers by “sva” R package. Then TCGA and additional cohorts were appointed as the training sets and testing sets separately. For each cancer the training set was used to establish necroptosis-related risk model by the least absolute shrinkage and selection operator (LASSO) regression, employing “glmnet” R package, with fivefold cross-validation applied to optimize the model. Patients were classified into low- and high-risk groups according to the median risk score of training set. Kaplan–Meier analysis of OS and the risk groups was conducted. To assess the predictive efficiency of the risk model, time-dependent receiver operating characteristic (ROC) curves of 1, 3, 5-years were made using “survivalROC” R package. Uni- and multi-variate survival analyses were employed to examine whether the risk score could independently affect patients’ prognosis. Model genes expression heat maps were constructed with the increase of risk score by “pheatmap” R package, and some clinical factors between patients from low- and high-risk groups were also compared by the use of Fisher’s exact test. Principal Component Analysis (PCA), t-distributed Stochastic Neighbor Embedding (t-SNE) and Uniform Manifold Approximation and Projection (UMAP) were carried out to verify the risk-group assignments according to the model genes expression data, where “stats”, “Rtsne” and “umap” R packages were used. Distribution of patients’ risk score and survival state was also analyzed. The same procedures were performed in the testing sets.

### Gene set enrichment analyses (GSEA)

In both training and testing sets, GSEA was conducted between low- and high-risk groups by “limma”, “org.Hs.eg.db”, “clusterProfiler”, “DOSE” and “enrichplot” R packages, with “kegg.v7.4.symbols” and “go.v7.4.symbols” downloaded from the MSigDB database. |Normalized enrichment score (NES)| > 1.5 and adjusted p-value < 0.05 were used as the screening criteria.

### Investigation of tumor immune microenvironment

Five algorithms were applied to assess immune infiltration status of each patient in both training and testing sets, namely, CIBERSORT, EPIC, MCPcounter, ssGSEA and ESTIMATE. Then, the immune infiltration level was compared between patients from low- and high-risk groups with Wilcoxon signed-rank test. The Spearman’s correlation analysis of risk score and immune score, stromal score as well as ESTIMATE score was also conducted. Then, we compared tumor mutational burden (TMB) and microsatellite instability (MSI) between the patients from the two risk groups with Wilcoxon signed-rank test, and investigated the relationship of risk score and TMB as well as MSI using Spearman’s correlation analysis. In addition, we explored whether there existed a correlation of risk score and immune related genes expression with Pearson correlation analysis, including immunoinhibitor genes, immunostimulator genes, Major Histocompatibility Complex (MHC) genes, chemokine genes and chemokine receptor genes. The corresponding genes were acquired from TISIDB (http://cis.hku.hk/TISIDB/index.php).

### Analysis of gene mutation

Somatic mutation data based on “VarScan2” software was acquired for TCGA samples. Then, we made oncoplots to show the mutation status of the top 20 most frequently mutated genes in low- and high-risk groups, with “maftools” R package. The mutation rate of the top 20 genes was compared by Fisher’s exact test.

### Drug sensitivity analysis

We downloaded the gene expression and z-score matrix from CellMiner (https://discover.nci.nih.gov/cellminer/home.do) and calculated the risk score of each sample according to the genes and corresponding coefficient of the different cancers’ risk model. Then, we investigated whether there existed any correlation of risk score and the sensitivity of Food and Drug Administration (FDA)-approved drugs with Pearson correlation analysis.

## Results

### Identification of prognostic DENGs in TCGA-cancers

**Fig. 1:**
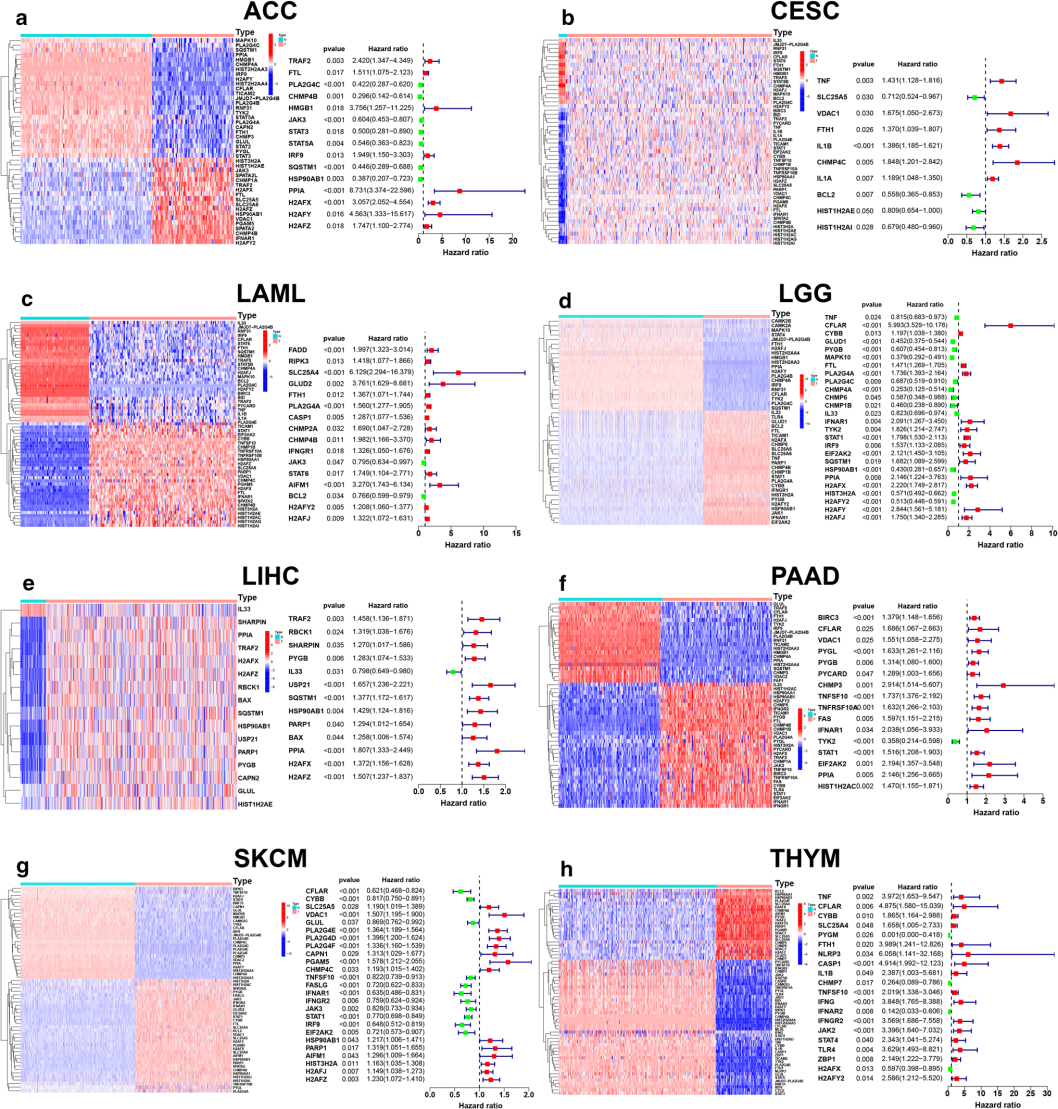
Identification of differentially expressed necroptosis-related genes (DENGs) and the investigation of their prognostic effect. Top 8 cancers with largest number of prognostic DENGs were chosen. The heat maps and forest plots showed the expression state and the prognostic effect of DENGs in adrenocortical carcinoma (ACC) (**a**), cervical squamous cell carcinoma endocervical adenocarcinoma (CESC) (**b**), acute myeloid leukemia (LAML) (**c**), brain lower grade glioma (LGG) (**d**), liver hepatocellular carcinoma (LIHC) (**e**), pancreatic adenocarcinoma (PAAD) (**f**), skin cutaneous melanoma (SKCM) (**g**) and thymoma (THYM) (**h**). |log2 (fold change)| > 1 and false discovery rate (FDR) < 0.05 were used as the screening criteria. Logrank p value and hazard ratio were presented beside each forest plot

**Fig. 2:**
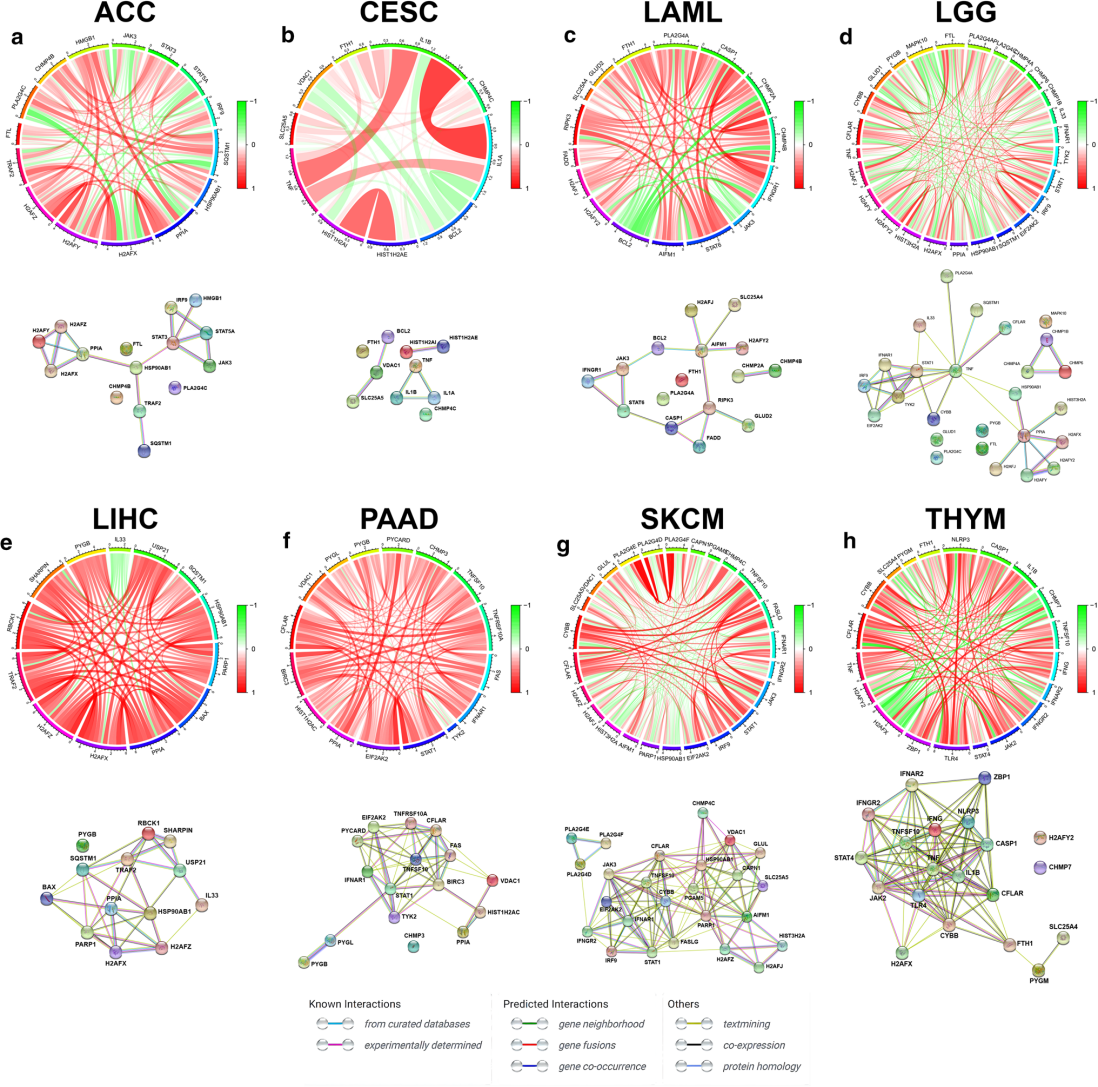
Correlation among prognostic DENGs. Chord diagrams and protein protein interaction networks (**a**–**h**) showed the correlation among the prognostic DENGs at mRNA and protein level in the eight cancers. The width and color of the lines between genes in chord diagrams represents the Pearson correlation coefficients and the sources of the protein-interactions were denoted with lines of distinct colors

As shown in Fig. 1, there were eight types of cancer with at least ten prognostic DENGs, namely, ACC, CESC, LAML, LGG, LIHC, PAAD, SKCM and THYM. The situation of other cancers was shown in Fig. S1, and no prognostic DENGs was found in colon adenocarcinoma (COAD) (d), stomach adenocarcinoma (STAD) (t), thyroid carcinoma (THCA) (u) and uterine corpus endometrial carcinoma (UCEC) (v). Notably, there were no DENGs observed in SARC. We also revealed the correlation between the prognostic DENGs in the eight cancers at both transcription and translation level (Fig. 2).

### Tumor classification

**Fig. 3:**
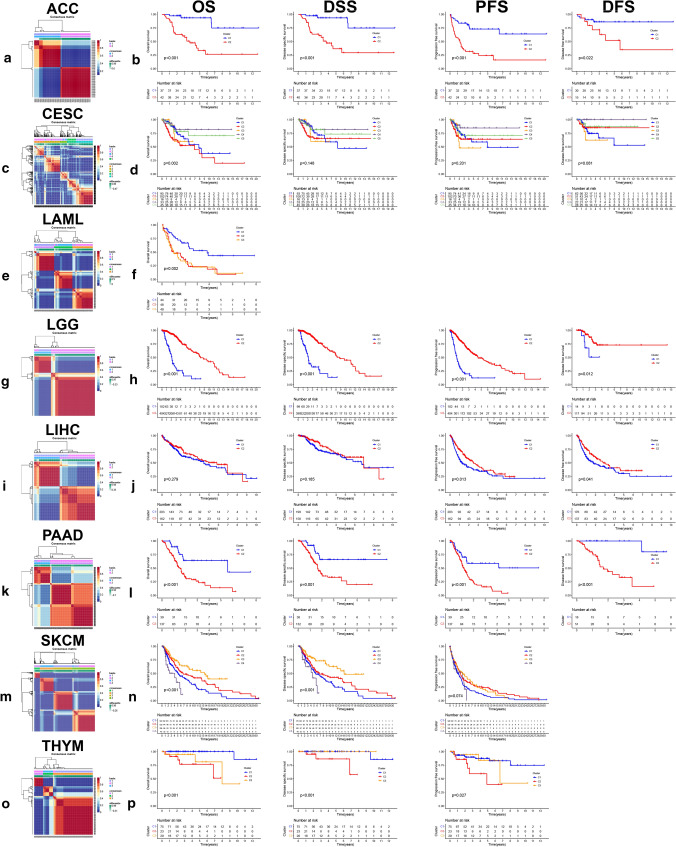
Non-negative matrix factorization (NMF) classification based on prognostic DENGs. The NMF consensus matrix heat maps based on optimal K value showed the classification status of ACC (**a**), CESC (**c**), LAML (**e**), LGG (**g**), LIHC (**i**), PAAD (**k**), SKCM (**m**) and THYM (**o**). Kaplan–Meier plots (**b**, **d**, **f**, **h**, **j**, **l**, **n**, **p**) showed the relationship of different clusters and overall survival (OS), disease specific survival (DSS), progression free survival (PFS) as well as disease free survival (DFS) in the eight cancers, with logrank p value marked in the graphs

We used NMF to classify cancer patients into different subgroups according to the expression profiles of the prognostic DENGs. NMF rank survey with multiple parameters and the consensus matrix heat maps were displayed at K value from 2 to 10 for ACC, CESC, LAML, LGG, LIHC, PAAD, SKCM and THYM (Fig. S2). The optimal K value was chosen for each cancer and the corresponding classification was shown (Fig. 3a, c, e, g, i, k, m, o). Notably, there existed significant difference of OS among the subgroups in all cancers except for LIHC (Fig. 3b, d, f, h, j, l, n, p).

### LASSO regression risk models

**Fig. 4:**
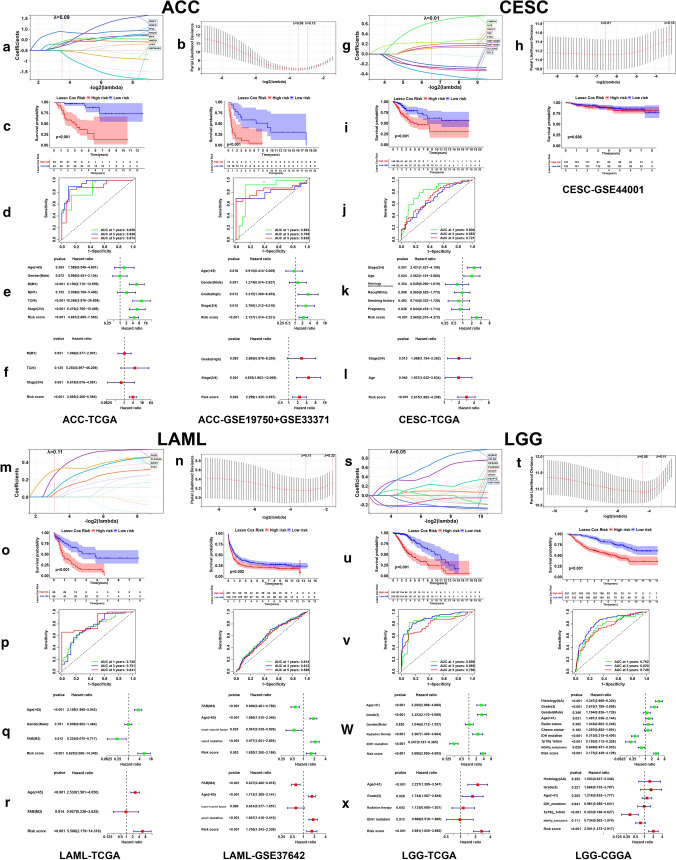
Risk model construction and validation based on prognostic DENGs in ACC, CESC, LAML and LGG. LASSO coefficient spectrum of the selected genes (**a**, **g**, **m**, **s**) and the fivefold cross-validation (**b**, **h**, **n**, **t**) for variable selection were shown. Kaplan–Meier plots (**c**, **i**, **o**, **u**) showed the OS difference between patients from low- and high-risk groups sorted by median risk score of the training set, with logrank p value marked in the graphs. Time-dependent receiver operating characteristic (ROC) curves of 1, 3, 5-years (**d**, **j**, **p**, **v**) showed the predictive efficiency of the risk model, with area under curve (AUC) values noted in the graphs. The forest plots showed the results of univariate (**e**, **k**, **q**, **w**) and multivariate (**f**, **l**, **r**, **x**) survival analyses

**Fig. 5:**
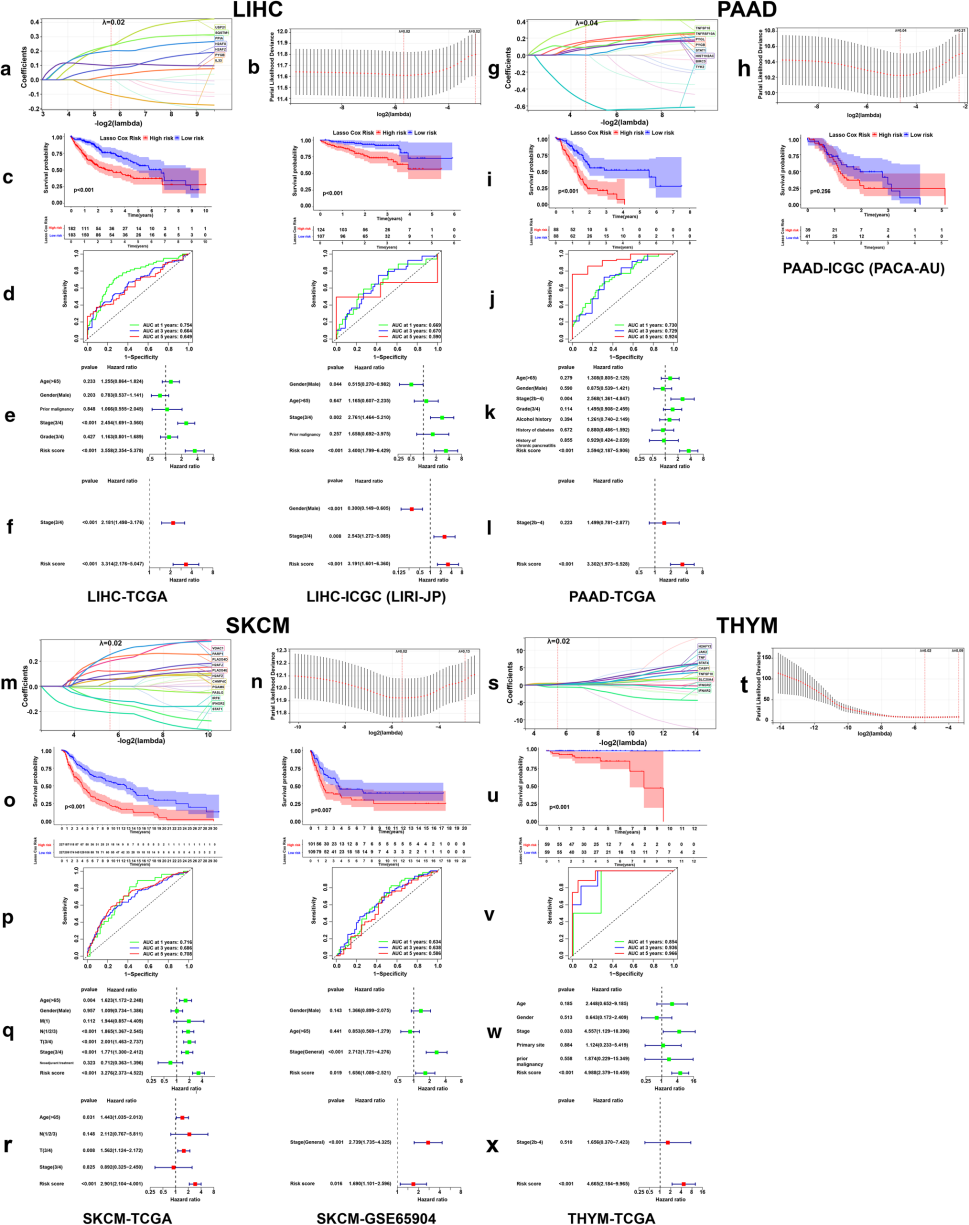
Risk model construction and validation based on prognostic DENGs in LIHC, PAAD, SKCM and THYM. LASSO coefficient spectrum of the selected genes (**a**, **g**, **m**, **s**) and the fivefold cross-validation (**b**, **h**, **n**, **t**) for variable selection were shown. Kaplan–Meier plots (**c**, **i**, **o**, **u**), time-dependent ROC curves of 1, 3, 5-years (**d**, **j**, **p**, **v**) and forest plots (**e**, **f**, **k**, **l**, **q**, **r**, **w**, **x**) showed the prognostic effectiveness of the risk models

**Fig. 6:**
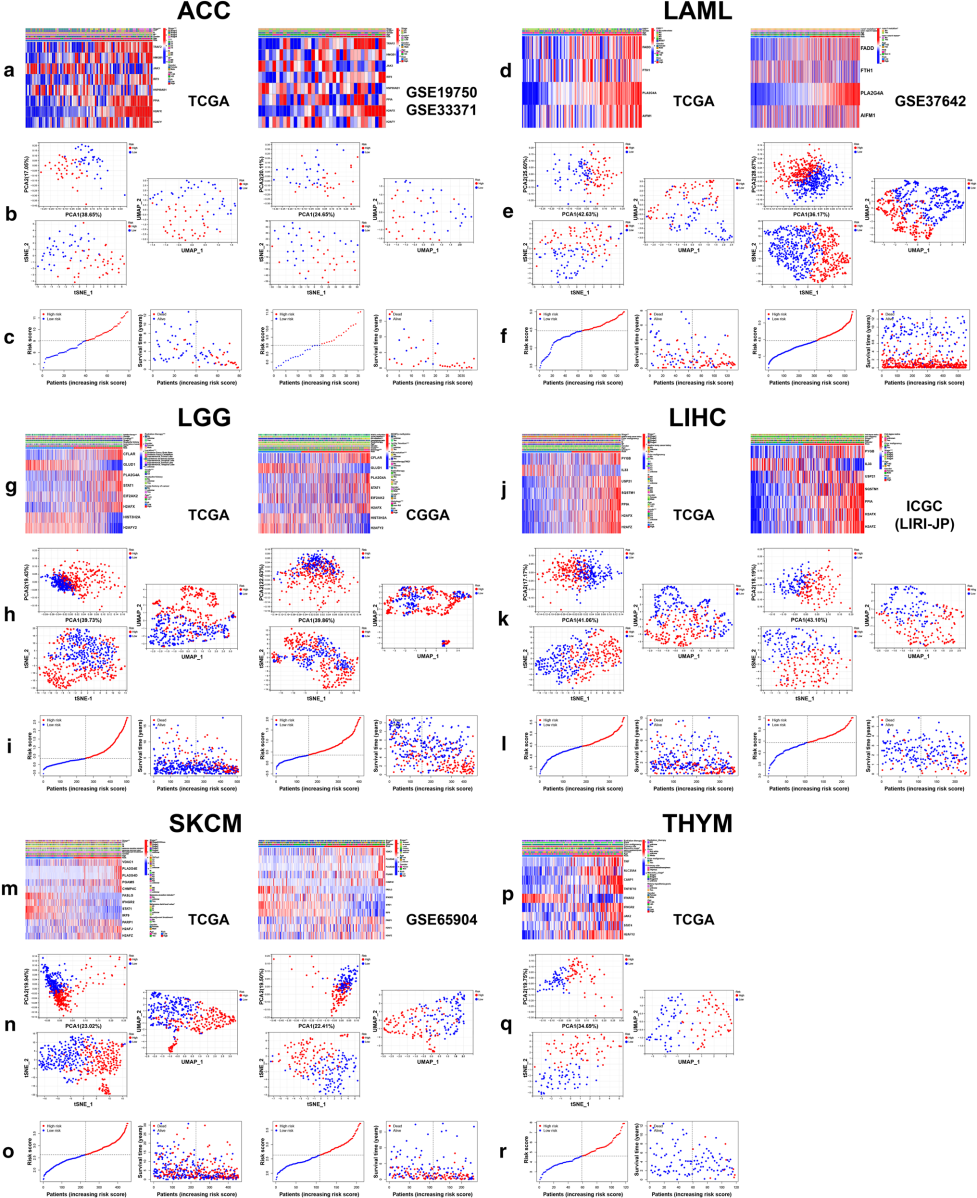
Model genes expression, dimensionality reduction analysis and distribution of risk score and survival state. The heat maps (**a**, **d**, **g**, **j**, **m**, **p**) showed the variation trend of model genes expression with the increase of risk score and the comparison of several clinical factors between low- and high-risk groups in the six selected cancers. Fisher’s exact test was used. *p < 0.05; **p < 0.01; ***p < 0.001. Principal Component Analysis (PCA), t-distributed Stochastic Neighbor Embedding (t-SNE) and Uniform Manifold Approximation and Projection (UMAP) (**b**, **e**, **h**, **k**, **n**, **q**) confirmed the stratification of patients into low- and high-risk clusters. The scatter diagrams (**c**, **f**, **i**, **l**, **o**, **r**) showed the condition of patients’ risk score and distribution of their survival time and state, with dotted line separating patients into low- and high-risk groups

The LASSO coefficient spectrum of the selected necroptosis-related genes for ACC, CESC, LAML, LGG, LIHC, PAAD, SKCM and THYM were shown in Figs. 4a, g, m, s and 5a, g, m, s. Figures 4b, h, n, t and 5b, h, n, t showed the fivefold cross-validation. The risk score calculation formulas of the eight cancers were shown in Supplementary file 2. In ACC, LAML, LGG, LIHC and SKCM, low-risk patients had obviously better OS compared with patients from high-risk group (Figs. 4c, o, u, 5c, o), and the time-dependent ROC curves of 1, 3 and 5 years in training and testing sets revealed the good efficiency of our risk models at predicting patients’ prognosis (Figs. 4d, p, v, 5d, p). The risk score could independently influence patients’ prognosis in both training and testing sets (Figs. 4f, r, x, 5f, r). However, In CESC and PAAD, we failed to observe the statistically significant difference of patients’ OS between low- and high-risk groups in the testing sets (Figs. 4i, 5i). We didn’t find a THYM cohort with sufficient prognostic information, so the analyses were only conducted in TCGA cohort (Fig. 5s–x). For ACC, LAML, LGG, LIHC, SKCM and THYM, the variation trend of model genes expression with the increase of risk score was shown, along with the comparison of some clinical factors between low- and high-risk groups (Fig. 6a, d, g, j, m, p). Dimensionality reduction analysis showed that the risk groups were largely in accordance with the two dimensional pattern of PCA, t-SNE and UMAP distribution, while in the testing set of LGG (CGGA cohort), the results were less satisfactory (Fig. 6b, e, h, k, n, q). With the increase of risk score, patients’ survival period was shortened and the number of deaths increased (Fig. 6c, f, i, l, o, r).

### GSEA result

**Fig. 7:**
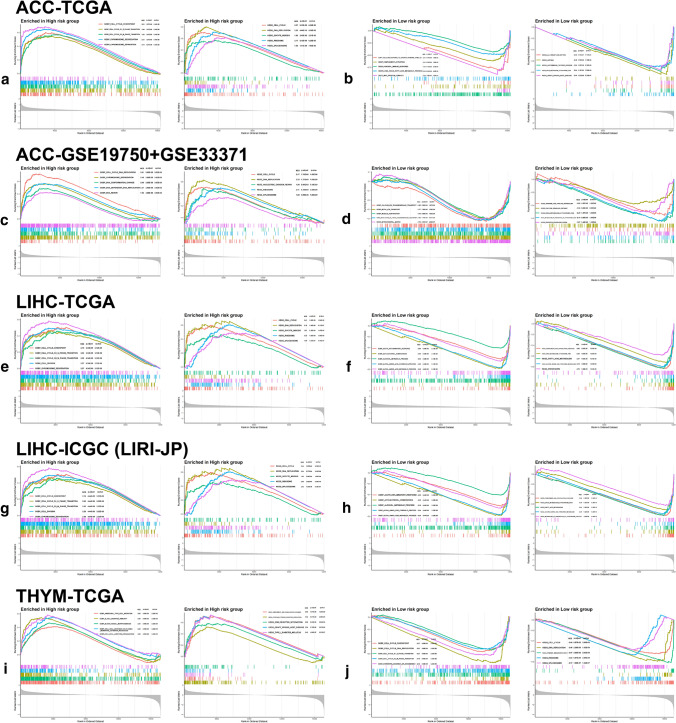
Gene Set Enrichment Analyses (GSEA) in ACC, LIHC and THYM. GSEA shows the top 5 gene ontology (GO) and Kyoto Encyclopedia of Genes and Genomes (KEGG) pathways enriched in low- and high-risk groups of ACC (**a**–**d**) and LIHC (**e**–**h**) at both training and testing sets. For THYM (**i**, **j**), GSEA was only conducted in TCGA cohort. Normalized enrichment score (NES), adjusted p-value and q-value were marked in the plots

**Fig. 8:**
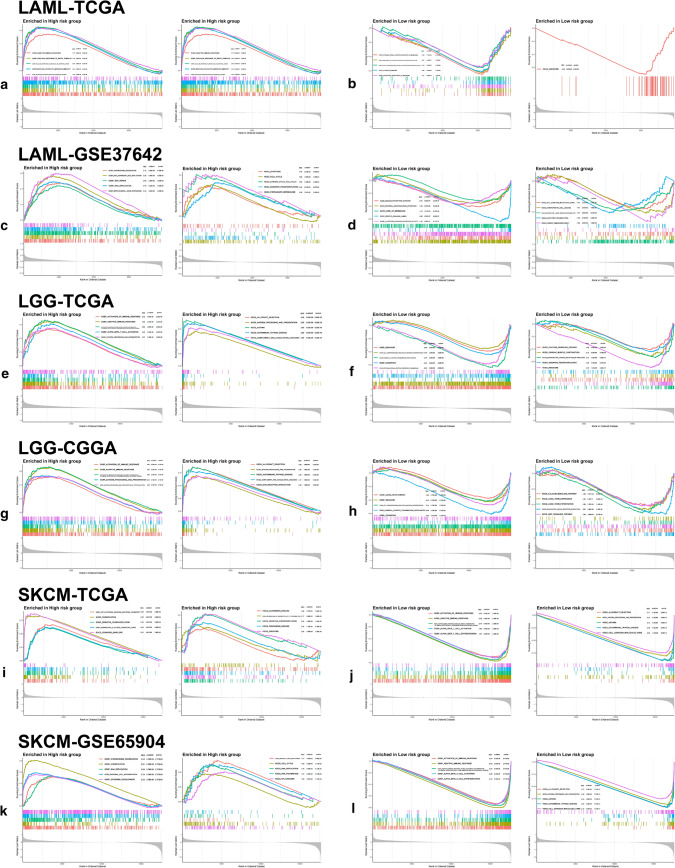
GSEA in LAML, LGG and SKCM. GSEA shows the top 5 GO and KEGG pathways enriched in low- and high-risk groups of LAML (**a**–**d**), LGG (**e**–**h**) and SKCM (**i**–**l**) at both training and testing sets. NES, adjusted p-value and q-value were marked in the plots

Gene Ontology (GO) and KEGG pathways related to the cell cycle were enriched in the high-risk group of ACC (Fig. 7a, c) and LIHC (Fig. 7e, g) no matter at training or testing sets, such as cell cycle checkpoint, cell cycle G1-S phase transition, cell cycle G2-M phase transition, chromosome segregation, DNA dependent DNA replication and splicesome, with similar situation observed in low-risk group of THYM (Fig. 7j). In addition, innate and adaptive immune-related pathways were enriched in LGG high-risk group (Fig. 8e, g) and SKCM low-risk group (Fig. 8j, l) no matter at training or testing sets, including activation of immune response, adaptive immune response, antigen presenting and presentation as well as complement and coagulation cascades. Surprisingly, in the analysis of LAML, we found visible enrichment discrepancies in high-risk group at training and testing sets, with immune-related or cell-circle-related pathways separately enriched in the two sets (Fig. 8a, c).

### Immune infiltration analysis of LGG and SKCM

**Fig. 9:**
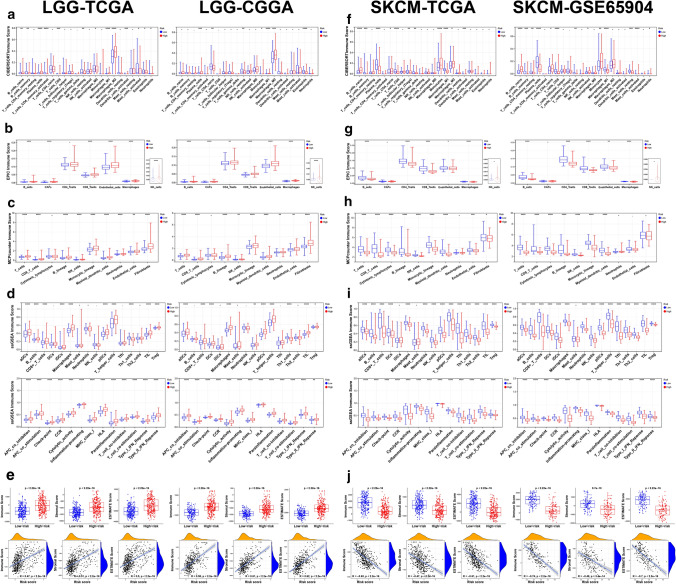
Immune infiltration analysis. The box plots and violin plots showed the difference of immune infiltration level and immune function between low- and high-risk groups of LGG and SKCM patients based on CIBERSORT (**a**, **f**), EPIC (**b**, **g**), MCPcounter (**c**, **h**) and ssGSEA (**d**, **i**), with Wilcoxon signed-rank test applied. *p < 0.05; **p < 0.01; ***p < 0.001; ****p < 0.0001. The scatter diagrams (**e**, **j**) showed the relationship between risk score and immune score, stromal score as well as ESTIMATE score, with Spearman’s correlation coefficient R value and p value marked in the plots

Based on the GSEA results above, we further explored whether there existed any immune infiltration difference between low- and high-risk groups in LGG and SKCM. According to five immune infiltration assessment algorithms, high-risk LGG patients and low-risk SKCM patients had higher level of immune infiltration and function at both training and testing sets, which accorded with the GSEA enrichment results. For LGG patients, the infiltration level of B cells, plasma cells, CD8+ T cells, macrophages, endothelial cells, cancer-associated fibroblasts (CAFs) and dendritic cells was higher in high-risk group (Fig. 9a–d), while the situation of NK cells (Fig. 9a–d) and regulatory T (Treg) cells (Fig. 9a, d) was different between the various algorithms. For SKCM patients, the infiltration level of B cells, plasma cells, CD8+ T cells, CD4+ T cells (Th1 cells, Th2 cells), gammadelta T cells, macrophages, endothelial cells, dendritic cells, follicular helper T (Tfh) cells and Treg cells was higher in low-risk group (Fig. 9f–i). As shown in Fig. 9e, immune score, stromal score and ESTIMATE score were higher in LGG patients from high-risk group at both training and testing sets, which also positively correlated with the patients’ risk score. For SKCM patients, the results were opposite (Fig. 9j).

**Fig. 10:**
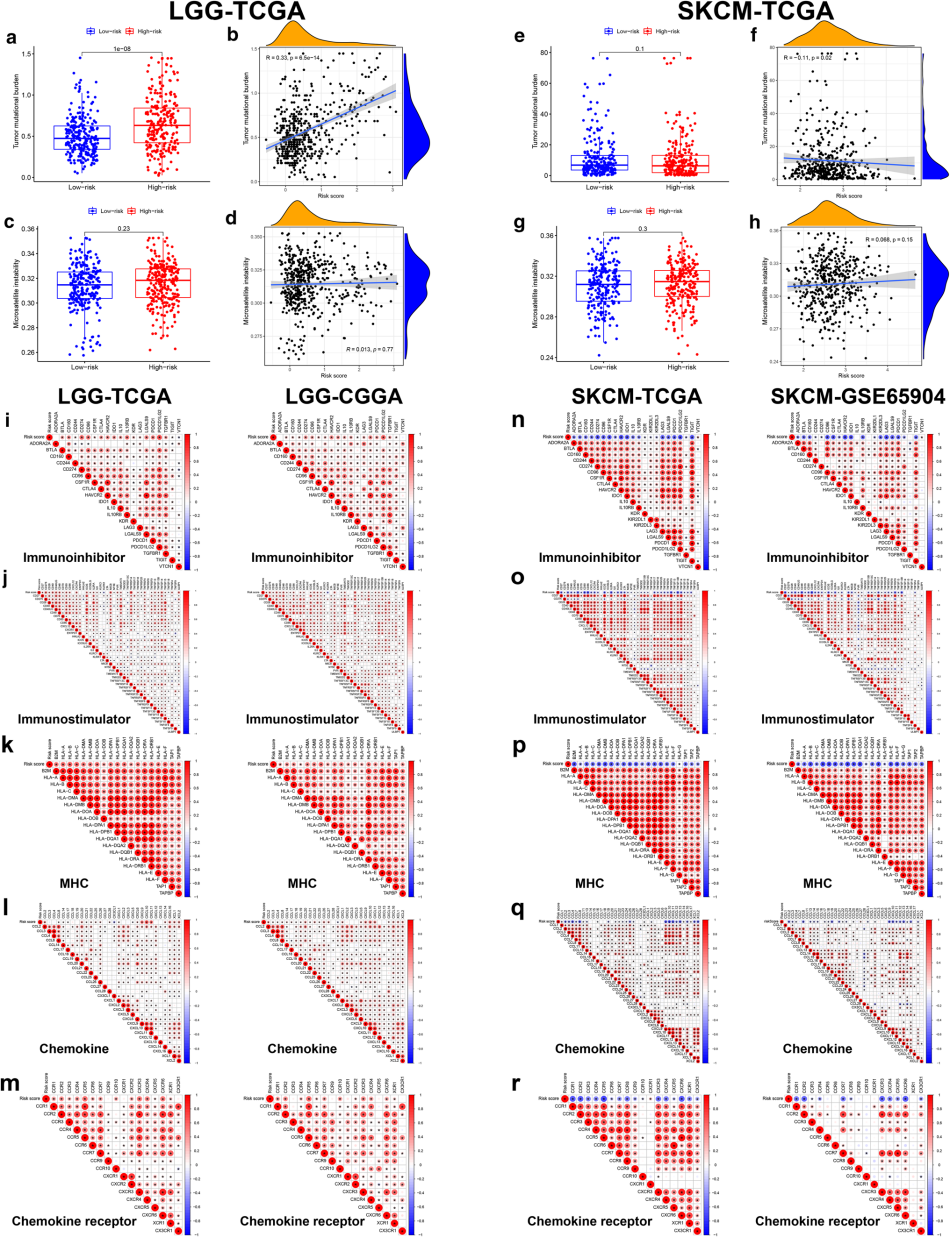
Tumor mutational burden (TMB), microsatellite instability (MSI) and immune-related genes expression analysis. Bar graphs showed the comparison of TMB (**a**, **e**) and MSI (**c**, **g**) between low- and high-risk groups and scatter diagrams showed the correlation between TMB (**b**, **f**) or MSI (**d**, **h**) and the risk score of LGG and SKCM patients. Wilcoxon signed-rank test p value and Spearman’s correlation coefficient R value as well p value were marked in the graphs. The correlations between risk score and the expression of immunoinhibitor genes (**i**, **n**), immunostimulator genes (**j**, **o**), MHC genes (**k**, **p**), chemokine genes (**l**, **q**) as well as chemokine receptor genes (**m**, **r**) were shown, with “*” representing Pearson correlation p value < 0.05

Then, we took TMB and MSI into consideration and found that high-risk LGG patients possessed higher TMB level (Fig. 10a), and TMB increased with risk score (Fig. 10b). Next, we explored the relationship of risk score and the gene expression of immunoinhibitors, immunostimulators, MHCs, chemokines and chemokine receptors. As shown in Fig. 10i–m, the expression of most immune-related genes positively correlated with risk score of LGG patients in both training and testing sets, while the results were opposite for SKCM patients (Fig. 10n–r).

### Gene mutation status

**Fig. 11:**
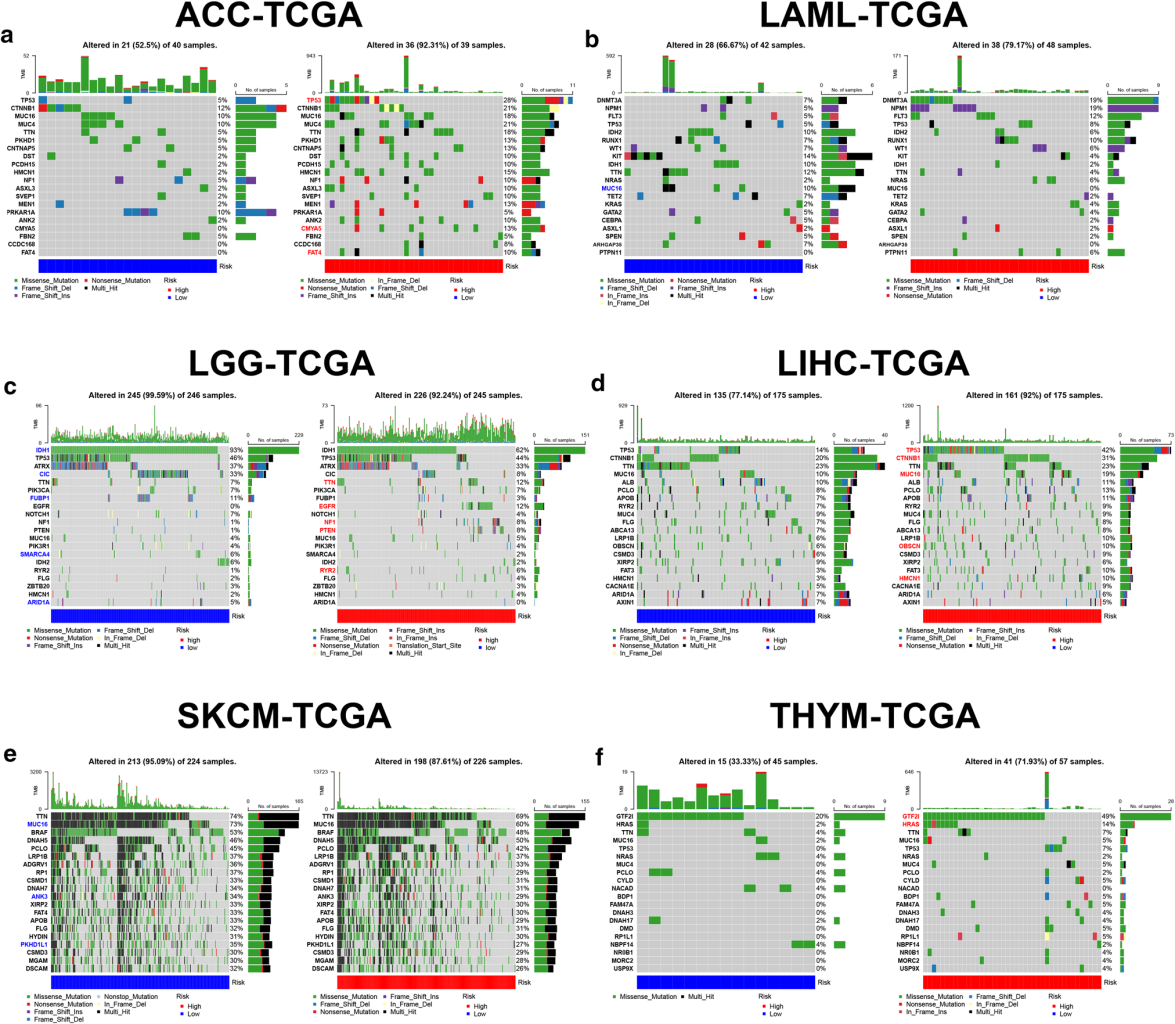
Gene mutation status in low- and high-risk groups. The oncoplots showed the mutation status of the top 20 most frequently mutated genes of ACC (**a**), LAML (**b**), LGG (**c**), LIHC (**d**), SKCM (**e**) and THYM (**f)** at low- and high-risk groups, with different colors referring to gene mutation types. The mutation rate of each gene between the two risk-groups was compared by Fisher’s exact test, and the genes with higher mutation rate in low- or high-risk groups were highlighted by blue or red color accordingly

We explored gene mutation status between low- and high-risk groups in TCGA cohorts of ACC, LAML, LGG, LIHC, SKCM and THYM, and screened out the top 20 genes with the highest mutation frequency. Higher mutation rate of tumor protein p53 (TP53) occurred in ACC and LIHC patients from high-risk group (Fig. 11a, d). For LAML and SKCM patients from low-risk group and LIHC patients form high-risk group, higher mutation rate of mucin 16, cell surface associated (MUC16) was observed (Fig. 11b, d, e). In addition, isocitrate dehydrogenase (NADP(+)) 1 (IDH1), capicua transcriptional repressor (CIC), far upstream element binding protein 1 (FUBP1), SWI/SNF related, matrix associated, actin dependent regulator of chromatin, subfamily a, member 4 (SMARCA4) and AT-rich interaction domain 1A (ARID1A) were more likely to mutate in LGG patents from low-risk group. However, higher mutation rate of titin (TTN), epidermal growth factor receptor (EGFR), neurofibromin 1 (NF1), phosphatase and tensin homolog (PTEN) and ryanodine receptor 2 (RYR2) was found in high-risk LGG patents (Fig. 11c). The mutations of general transcription factor IIi (GTF2I) and HRas proto-oncogene, GTPase (HRAS) were more common in high-risk THYM patients (Fig. 11f).

### Correlation between risk score and drug sensitivity

**Fig. 12:**
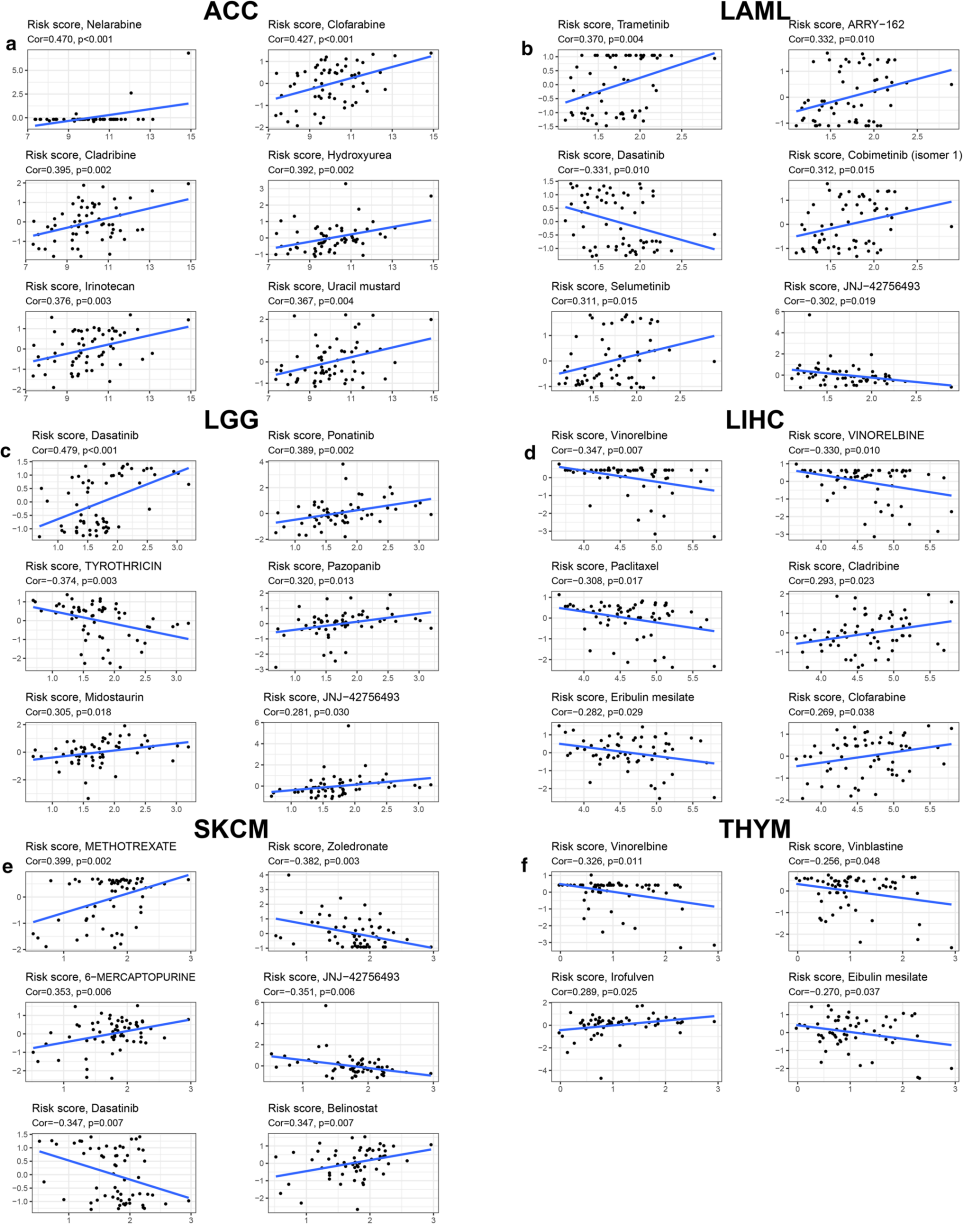
Drug sensitivity exploration. We calculated the risk score of each CellMiner sample according to the genes and coefficient of the risk models of the six cancers. The scatter diagrams showed the correlation between risk score and sensitivity (z-score) of Food and Drug Administration (FDA)-approved drugs in ACC (**a**), LAML (**b**), LGG (**c**), LIHC (**d**), SKCM (**e**) and THYM (**f**), with Pearson correlation coefficient (Cor) and p value marked above the graphs

Finally, we paid attention to the drug selection. As shown in Fig. 12a, d, with the increase of risk score, ACC and LIHC may be more sensitive to adenine nucleotide analogues, such as nelarabine, clofarabine and cladribine. For high-risk LGG and LAML/SKCM with low-risk score, dasatinib was perhaps a good choice (Fig. 12b, c, e). For THYM, the irofulven sensitivity positively correlated with risk score, but a negative correlation was detected between the sensitivity of vinorelbine, vinblastine as well as eribulin mesilate and risk score (Fig. 12f).

## Discussion

Necroptosis is a novel programmed cell death mode independent on caspase, with increasing evidence of anti-tumor effects discovered in recent years. As we know, traditional chemotherapeutic agents usually induced cell apoptosis to exert anti-tumor effects [26]. However, tumor cells are inherently anti-apoptotic. In spite of the prevalence of heterogeneity in various tumors, there’s a high possibility that the subpopulation of tumor cells with greater anti-apoptotic selection superiority will gradually clone and govern the entire tumor as the treatment proceeds. Therefore, drug resistance has become a common fact during clinical practice, and tumors which relapse or progress after treatment are extremely difficult to deal with [26]. Thus, it became a natural idea to induce other types of cell death for drug-resistant tumors, and alternative choices mainly included ferroptosis, pyroptosis as well as necroptosis [27]. Numerous studies have proven that the transition of apoptosis to necroptosis or the direct induction of necroptosis could make for overcoming drug resistance and inhibiting tumor development for various cancers, such as acute myeloid leukemia [28, 29], breast cancer [30], osteosarcoma [31], nasopharyngeal carcinoma [32], prostate cancer [33, 34] and colon cancer [35, 36].

In this study, based on TCGA and GTEx data, we identified eight types of cancer with the highest number of prognostic DENGs and for the first time sorted ACC, CESC, LAML, LGG, LIHC, PAAD, SKCM and THYM patients into different subgroups based on necroptosis-related genes. Kaplan–Meier analysis of four follow-up endpoints showed that the classification was excellent in distinguishing patients’ OS in all cancers above except for LIHC. Then, the risk models were set up. Unfortunately, the risk models didn’t work at testing sets of CESC and PAAD, but we do find a method to efficiently distinguish patients’ OS in ACC, LAML, LGG, LIHC and SKCM. The testing set of LAML (GSE37642) lacked M3-subtype patients and the testing set of LGG (CGGA) only consisted of Asian patients, so there existed some intrinsic discrepancies between TCGA cohorts (used as training set) and these testing sets. This might cause the inconsistency of AUC values between training and testing set. Notably, among these five cancer types, ACC is relatively less studied. As a rare malignancy with great complexity, the 5-year DFS rate of ACC was only about 30%, and there still existed many therapeutic challenges [37, 38]. Due to the heterogeneity of ACC, the prognostic efficiency of the most widely accepted TNM staging was inevitably limited [39]. Thus, it is necessary to seek new risk factors for ACC patients. Our ACC risk model based on necroptosis-related genes has good predictive ability for patient’ survival, which might provide meaningful references for patients’ prognosis in the future clinical practice.

Although kinds of immunotherapies have achieved remarkable success in cancer treatment, only limited number of patients could exhibit long-lasting anti-tumor response, where tumor immune infiltration status played a significant role [40]. Identification of cancer patients with abundant infiltration of immune cells is of great importance to screen out potential candidates for immunotherapy. Our GSEA results of SKCM and LGG cohorts highlighted immune-related GO and KEGG pathways in low- and high- risk groups, which along with results of the estimated immune infiltration level based on five algorithms could contribute to the distinction of “cold” and “hot” tumors.

As we know, immunotherapies have not acquired satisfactory results in glioma patients in recent years, including adoptive lymphocyte transfer, tumor associated vaccine, viral-based therapy and ICIs, where T-cell exhaustion played a dominant role, and tumor heterogeneity, blood brain barrier as well as lack of immune organs in central nerve systems also shared the blame [41]. Although there is a higher CD8+ T cells infiltration level in high-risk LGG patients, we failed to observe the difference of cytotoxic lymphocytes between the two risk groups according to MCPcounter. Noteworthy is the infiltration level of M2 macrophages and CAFs is higher in high-risk LGG patients. Recent studies have revealed the fact that M2 macrophages played a vital part in the development of glioma by promoting tumor invasion and metastasis, facilitating tumor stemness as well as suppressing immunity of the tumor area and the whole body [42, 43]. CAFs were involved in tumor cell replication, angiogenesis, chemotherapy insensitivity and the suppression of CD8+ T cell function [44, 45]. M2 macrophages and CAFs have been considered as promising therapeutic targets by number of studies [44–46], and high-risk LGG patients perhaps benefit from the agents which inhibit M2 macrophages or CAFs.

Unlike the situation in LGG, the infiltration level of immune cells widely known for suppressing tumor development is higher in low-risk SKCM patients, including CD8+ T cells, Th1 cells and M1 macrophages. According to the correlation analysis of risk score and immune-related gene expression, SKCM patients from low-risk group also possessed a higher gene expression level of plenty of immunosuppressive molecules, some of which were identified as immune checkpoints and their therapeutic potential has been proven by numerous studies. ICIs were initially studied and applied for the clinical application in melanoma, and Ipilimumab, targeting cytotoxic T-lymphocyte-associated protein 4 (CTLA4), is the first drug in history to significantly prolong the survival period of patients with this highly malignant tumor [47]. Programmed cell death protein 1 (PD-1) antibody was also approved for the treatment of advanced melanoma by FDA in the year of 2014 and phase 3 clinical trial of Relatlimab, targeting lymphocyte-activation gene 3 (LAG-3), has met its primary endpoint of PFS, which may offer new hope for SKCM patients in the future. It needs to be mentioned that there existed a higher mutation rate of MUC16 in low-risk SKCM patients. MUC16, also known as carbohydrate antigen 125 (CA125), ranks third in the list of gene mutation frequency of cancers, whose mutation occurs most frequently in SKCM [48]. The study also showed that MUC16-mutated melanoma patients treated with ICIs had significantly longer OS. Given that our study could help to recognize SKCM patients with higher level of immune infiltration and immune-checkpoint genes expression as well as higher MUC16 mutation rate, it is reasonable to believe that low-risk SKCM patients are more likely to benefit from ICIs treatment.

Although we failed to find a cohort to check the predictive ability of prognosis in the THYM risk model, there were still some results which could arouse our attention. First, the nine-genes risk model successfully assigned all death cases into high-risk group, and the following time-dependent ROC analysis exhibited an excellent predictive ability of the model with 1, 3, 5-year OS area under the ROC curve up to 0.854, 0.936 and 0.966. Regardless of the application of which dimensionality reduction method, the cases could be obviously divided into low- and high-risk clusters. Thymoma has a low incidence and favorable prognosis, so the associated studies are relatively limited compared with other common or highly malignant tumors. For patients classified as high-risk, their review period perhaps needs to be shortened so that the tumor progression can be detected and treated in time.

For THYM, it is still controversial whether adjuvant radiotherapy or chemotherapy should be applied after surgery. According to our result, some of the patients classified as high-risk might be the potential candidates for postoperative adjuvant therapies. We noticed a decline in the sensitivity of tumor cells to vincaleukoblastinum drugs with the increase of risk score based on THYM risk model. However, irofulven exhibited anti-tumor activity in cells with high risk score, which is a kind of cytotoxic drug proven to be an effective agent for tumors with DNA repair deficiency by several studies [49, 50]. This finding may provide some useful information for the clinical chemotherapy of THYM. In addition, we noticed the mutation rate of GTF2I in the high-risk patients was about twice as high as that in low-risk patients. Researchers have found that there existed a high mutation rate of GTF2I in indolent thymomas, which was extremely rare in aggressive thymomas and thymic carcinomas [51]. Mutant GTF2I, identified as a novel tumorigenic driver, can promote growth, proliferation and transformation of epithelial cell as well as alter glucose and lipid metabolism [51, 52], and whether it could work as a therapeutic target requires further research.

## Conclusions

In summary, this is the first study to comprehensively investigate the genes of necroptosis pathway in all TCGA cancers. We conducted NMF to classify ACC, CESC, LAML, LGG, PAAD, SKCM and THYM patients into subgroups with different prognosis. The risk model based on necroptosis-related genes can effectively predict the prognosis of ACC, LAML, LGG, LIHC, SKCM and THYM patients. The risk score contributes to the identification of immune infiltration level for LGG and SKCM patients, which could help to screen out the potential candidates who might benefit from immunotherapy. Genetic mutation status and drug sensitivity were also different for patients from different risk groups, which may offer meaningful information for the future clinical practice.

## Supplementary Information

Below is the link to the electronic supplementary material.

Supplementary file 1 (DOCX 34 KB)

Supplementary file 2 (DOCX 16 KB)

Supplementary file 3 Figure S1. Differentially expressed necroptosis-related genes (DENGs) and survival analysis in other cancers. The heat maps and forest plots showed the expression state and the prognostic effect of the DENGs in bladder urothelial carcinoma (BLCA) (**a**), breast invasive carcinoma (BRCA) (**b**), cholangiocarcinoma (CHOL) (**c**), colon adenocarcinoma (COAD) (**d**), lymphoid neoplasm diffuse large B-cell lymphoma (DLBC) (**e**), esophageal carcinoma (ESCA) (**f**), glioblastoma multiforme (GBM) (**g**), head and neck squamous cell carcinoma (HNSC) (**h**), kidney chromophobe (KICH) (**i**), kidney renal clear cell carcinoma (KIRC) (**j**), kidney renal papillary cell carcinoma (KIRP) (**k**), lung adenocarcinoma (LUAD) (**l**), lung squamous cell carcinoma (LUSC) (**m**), ovarian serous cystadenocarcinoma (OV) (**n**), pheochromocytoma and paraganglioma (PCPG) (**o**), prostate adenocarcinoma (PRAD) (**p**), rectum adenocarcinoma (READ) (**q**), testicular germ cell tumors (TGCT) (**r**), uterine carcinosarcoma (UCS) (**s**), stomach adenocarcinoma (STAD) (**t**), thyroid carcinoma (THCA) (**u**), uterine corpus endometrial carcinoma (UCEC) (**v**). |log2 (fold change)| > 1 and false discovery rate (FDR) < 0.05 were used as the screening criteria for the detection of DENGs between tumor and normal tissues. Logrank p value and hazard ratio were presented beside each forest plot (TIF 9868 KB)

Supplementary 4 Figure S2. Comprehensive correlation coefficient and the consensus matrix heat maps of NMF analysis. The NMF rank survey and the consensus matrix heat maps were exhibited with K values ranking from 2 to 10 in adrenocortical carcinoma (ACC) (**a**), cervical squamous cell carcinoma endocervical adenocarcinoma (CESC) (**b**), acute myeloid leukemia (LAML) (**c**), brain lower grade glioma (LGG) (**d**), liver hepatocellular carcinoma (LIHC) (**e**), pancreatic adenocarcinoma (PAAD) (**f**), skin cutaneous melanoma (SKCM) (**g**) and thymoma (THYM) (**h**) (TIF 8507 KB)

## Data Availability

The datasets used in this article can be acquired from the internet. They can be downloaded from the corresponding open databases mentioned in this article.

## Abbreviations

TCGA: The Cancer Genome Atlas
GTEx: Genotype-Tissue Expression
GEO: Gene Express Omnibus
ICGC: International Cancer Genome Consortium
CGGA: Chinese Glioma Genome Atlas
ACC: Adrenocortical carcinoma
BLCA: Bladder urothelial carcinoma
BRCA: Breast invasive carcinoma
CESC: Cervical squamous cell carcinoma and endocervical adenocarcinoma
CHOL: Cholangiocarcinoma
COAD: Colon adenocarcinoma
DLBC: Lymphoid neoplasm diffuse large B-cell lymphoma
DLBC: Lymphoid neoplasm diffuse large B-cell lymphoma
ESCA: Esophageal carcinoma
GBM: Glioblastoma multiforme
HNSC: Head and neck squamous cell carcinoma
KICH: Kidney chromophobe
KIRC: Kidney renal clear cell carcinoma
KIRP: Kidney renal papillary cell carcinoma
LAML: Acute myeloid leukemia
LGG: Brain lower grade glioma
LIHC: Liver hepatocellular carcinoma
LUAD: Lung adenocarcinoma
LUSC: Lung squamous cell carcinoma
MESO: Mesothelioma
OV: Ovarian serous cystadenocarcinoma
PAAD: Pancreatic adenocarcinoma
PCPG: Pheochromocytoma and paraganglioma
PRAD: Prostate adenocarcinoma
READ: Rectum adenocarcinoma
SARC: Sarcoma
SKCM: Skin cutaneous melanoma
STAD: Stomach adenocarcinoma
TGCT: Testicular germ cell tumors
THCA: Thyroid carcinoma
THYM: Thymoma
UCEC: Uterine corpus endometrial carcinoma
UCS: Uterine carcinosarcoma
UVM: Uveal melanoma
FADD: Fas-associated protein with death domain
TNF-α: Tumor necrosis factor α
NCCD: Nomenclature Committee on Cell Death
TNFR1: Tumor necrosis factor receptor 1
TRADD: TNF receptor 1-associated death domain protein
TRAF2: Tumor necrosis factor and receptor related factor 2
RIPK1: Receptor-interacting protein kinase 1
CIAP1/2: Cellular inhibitors of apoptosis 1 and 2
LUBAC: Linear ubiquitin Chain assembly complex
TGF-β: Transforming growth factor-beta
TAK1/TAB: TGF-β activated kinase 1/TGF-β activated kinase 1 binding protein
CYLD: Cylindromatosis
RIPK3: Receptor-interacting protein kinase 3
MLKL: Mixed lineage kinase domain-like
DAMPs: Damage associated molecular patterns
TAM: Tumor-associated macrophage
CXCL1: C-X-C motif chemokine ligand 1
KEGG: Kyoto Encyclopedia of Genes and Genomes
DENGs: Differentially expressed necroptosis-related genes
NMF: Non-negative matrix factorization
OS: Overall survival
DSS: Disease specific survival
PFS: Progression free survival
DFS: Disease free survival
LASSO: Least absolute shrinkage and selection operator
ROC: Receiver operating characteristic
PCA: Principal Component Analysis
t-SNE: T-distributed Stochastic Neighbor Embedding
UMAP: Uniform Manifold Approximation and Projection
GSEA: Gene Set Enrichment Analyses
TMB: Tumor mutational burden
MSI: Microsatellite instability
MHC: Major Histocompatibility Complex
FDA: Food and Drug Administration
GO: Gene Ontology
CAFs: Cancer-associated fibroblasts
Treg: Regulatory T
Tfh: Follicular helper T
TP53: Tumor protein p53
IDH1: Isocitrate dehydrogenase (NADP(+)) 1
CIC: Capicua transcriptional repressor
FUBP1: Far upstream element binding protein 1
SMARCA4: SWI/SNF related, matrix associated, actin dependent regulator of chromatin, subfamily a, member 4
ARID1A: AT-rich interaction domain 1A
TTN: Titin
EGFR: Epidermal growth factor receptor
NF1: Neurofibromin 1
PTEN: Phosphatase and tensin homolog
RYR2: Ryanodine receptor 2
MUC16: Mucin 16, cell surface associated
ANK3: Ankyrin 3
PKHD1L1: PKHD1 like 1
GTF2I: General transcription factor IIi
HRAS: HRas proto-oncogene, GTPase
CTLA4: Cytotoxic T-lymphocyte-associated protein 4
PD-1: Programmed cell death protein 1
LAG-3: Lymphocyte-activation gene 3
CA125: Carbohydrate antigen 125
