# Supplementary material for: Pan-cancer analysis of necroptosis-related gene signature for the identification of prognosis and immune significance
Source: Discov Oncol. 2022 Mar 21;13:17. doi: 10.1007/s12672-022-00477-2 (PMC8938586; doi:10.1007/s12672-022-00477-2)
Supplement: Supplementary file 1 — (DOCX 34 KB) [file 12672_2022_477_MOESM1_ESM.docx]

**Necroptosis-related genes list (KEGG-hsa04217)**

| Genes | Gene description |
| --- | --- |
| [TNF](https://www.genome.jp/entry/K03156) | tumor necrosis factor |
| [TNFRSF1A](https://www.genome.jp/entry/K03158) | TNF receptor superfamily member 1A |
| [TRADD](https://www.genome.jp/entry/K03171) | TNFRSF1A associated via death domain |
| TRAF2 | TNF receptor associated factor 2 |
| [TRAF5](https://www.genome.jp/entry/K09849) | TNF receptor associated factor 5 |
| RIPK1 | receptor interacting serine/threonine kinase 1 |
| [BIRC2](https://www.genome.jp/entry/K16060) | baculoviral IAP repeat containing 2 |
| [BIRC3](https://www.genome.jp/entry/K16060) | baculoviral IAP repeat containing 3 |
| XIAP | X-linked inhibitor of apoptosis |
| RBCK1 | RANBP2-type and C3HC4-type zinc finger containing 1 |
| RNF31 | ring finger protein 31 |
| [SHARPIN](https://www.genome.jp/entry/K20894) | SHANK associated RH domain interactor |
| [SPATA2L](https://www.genome.jp/entry/K17595) | spermatogenesis associated 2 like |
| [SPATA2](https://www.genome.jp/entry/K17595) | spermatogenesis associated 2 |
| CYLD | CYLD lysine 63 deubiquitinase |
| [FADD](https://www.genome.jp/entry/K02373) | Fas associated via death domain |
| CASP8 | caspase 8 |
| [CFLAR](https://www.genome.jp/entry/K04724) | CASP8 and FADD like apoptosis regulator |
| RIPK3 | receptor interacting serine/threonine kinase 3 |
| [CYBB](https://www.genome.jp/entry/K21421) | cytochrome b-245 beta chain |
| CAMK2A | calcium/calmodulin dependent protein kinase II alpha |
| CAMK2D | calcium/calmodulin dependent protein kinase II delta |
| CAMK2B | calcium/calmodulin dependent protein kinase II beta |
| CAMK2G | calcium/calmodulin dependent protein kinase II gamma |
| [SLC25A4](https://www.genome.jp/entry/K05863) | solute carrier family 25 member 4 |
| [SLC25A5](https://www.genome.jp/entry/K05863) | solute carrier family 25 member 5 |
| [SLC25A6](https://www.genome.jp/entry/K05863) | solute carrier family 25 member 6 |
| [SLC25A31](https://www.genome.jp/entry/K05863) | solute carrier family 25 member 31 |
| PPID | peptidylprolyl isomerase D |
| [VDAC1](https://www.genome.jp/entry/K05862) | voltage dependent anion channel 1 |
| [VDAC2](https://www.genome.jp/entry/K15040) | voltage dependent anion channel 2 |
| [VDAC3](https://www.genome.jp/entry/K15041) | voltage dependent anion channel 3 |
| GLUD2 | glutamate dehydrogenase 2 |
| GLUD1 | glutamate dehydrogenase 1 |
| GLUL | glutamate-ammonia ligase |
| PYGL | glycogen phosphorylase L |
| PYGM | glycogen phosphorylase, muscle associated |
| PYGB | glycogen phosphorylase B |
| MAPK8 | mitogen-activated protein kinase 8 |
| MAPK10 | mitogen-activated protein kinase 10 |
| MAPK9 | mitogen-activated protein kinase 9 |
| FTH1 | ferritin heavy chain 1 |
| [FTL](https://www.genome.jp/entry/K13625) | ferritin light chain |
| PLA2G4E | phospholipase A2 group IVE |
| PLA2G4A | phospholipase A2 group IVA |
| JMJD7-PLA2G4B | JMJD7-PLA2G4B readthrough |
| PLA2G4B | phospholipase A2 group IVB |
| PLA2G4C | phospholipase A2 group IVC |
| PLA2G4D | phospholipase A2 group IVD |
| PLA2G4F | phospholipase A2 group IVF |
| ALOX15 | arachidonate 15-lipoxygenase |
| CAPN1 | calpain 1 |
| CAPN2 | calpain 2 |
| SMPD1 | sphingomyelin phosphodiesterase 1 |
| MLKL | mixed lineage kinase domain like pseudokinase |
| PGAM5 | PGAM family member 5, mitochondrial serine/threonine protein phosphatase |
| DNM1L | dynamin 1 like |
| [NLRP3](https://www.genome.jp/entry/K12800) | NLR family pyrin domain containing 3 |
| [PYCARD](https://www.genome.jp/entry/K12799) | PYD and CARD domain containing |
| CASP1 | caspase 1 |
| [IL1B](https://www.genome.jp/entry/K04519) | interleukin 1 beta |
| [CHMP2A](https://www.genome.jp/entry/K12191) | charged multivesicular body protein 2A |
| [CHMP2B](https://www.genome.jp/entry/K12192) | charged multivesicular body protein 2B |
| [CHMP3](https://www.genome.jp/entry/K12193) | charged multivesicular body protein 3 |
| [RNF103-CHMP3](https://www.genome.jp/entry/K12193) | RNF103-CHMP3 readthrough |
| [CHMP4B](https://www.genome.jp/entry/K12194) | charged multivesicular body protein 4B |
| [CHMP4A](https://www.genome.jp/entry/K12194) | charged multivesicular body protein 4A |
| [CHMP4C](https://www.genome.jp/entry/K24782) | charged multivesicular body protein 4C |
| [CHMP6](https://www.genome.jp/entry/K12195) | charged multivesicular body protein 6 |
| [VPS4B](https://www.genome.jp/entry/K12196) | vacuolar protein sorting 4 homolog B |
| [VPS4A](https://www.genome.jp/entry/K12196) | vacuolar protein sorting 4 homolog A |
| [CHMP1B](https://www.genome.jp/entry/K12197) | charged multivesicular body protein 1B |
| [CHMP1A](https://www.genome.jp/entry/K12197) | charged multivesicular body protein 1A |
| [CHMP5](https://www.genome.jp/entry/K12198) | charged multivesicular body protein 5 |
| [CHMP7](https://www.genome.jp/entry/K15053) | charged multivesicular body protein 7 |
| TRPM7 | transient receptor potential cation channel subfamily M member 7 |
| [IL1A](https://www.genome.jp/entry/K04383) | interleukin 1 alpha |
| [IL33](https://www.genome.jp/entry/K12967) | interleukin 33 |
| [HMGB1](https://www.genome.jp/entry/K10802) | high mobility group box 1 |
| [TNFSF10](https://www.genome.jp/entry/K04721) | TNF superfamily member 10 |
| [TNFRSF10A](https://www.genome.jp/entry/K04722) | TNF receptor superfamily member 10a |
| [TNFRSF10B](https://www.genome.jp/entry/K04722) | TNF receptor superfamily member 10b |
| [FASLG](https://www.genome.jp/entry/K04389) | Fas ligand |
| [FAS](https://www.genome.jp/entry/K04390) | Fas cell surface death receptor |
| [FAF1](https://www.genome.jp/entry/K20703) | Fas associated factor 1 |
| [IFNA1](https://www.genome.jp/entry/K05414) | interferon alpha 1 |
| [IFNA2](https://www.genome.jp/entry/K05414) | interferon alpha 2 |
| [IFNA4](https://www.genome.jp/entry/K05414) | interferon alpha 4 |
| [IFNA5](https://www.genome.jp/entry/K05414) | interferon alpha 5 |
| [IFNA6](https://www.genome.jp/entry/K05414) | interferon alpha 6 |
| [IFNA7](https://www.genome.jp/entry/K05414) | interferon alpha 7 |
| [IFNA8](https://www.genome.jp/entry/K05414) | interferon alpha 8 |
| [IFNA10](https://www.genome.jp/entry/K05414) | interferon alpha 10 |
| [IFNA13](https://www.genome.jp/entry/K05414) | interferon alpha 13 |
| [IFNA14](https://www.genome.jp/entry/K05414) | interferon alpha 14 |
| [IFNA16](https://www.genome.jp/entry/K05414) | interferon alpha 16 |
| [IFNA17](https://www.genome.jp/entry/K05414) | interferon alpha 17 |
| [IFNA21](https://www.genome.jp/entry/K05414) | interferon alpha 21 |
| [IFNB1](https://www.genome.jp/entry/K05415) | interferon beta 1 |
| [IFNG](https://www.genome.jp/entry/K04687) | interferon gamma |
| [IFNAR1](https://www.genome.jp/entry/K05130) | interferon alpha and beta receptor subunit 1 |
| [IFNAR2](https://www.genome.jp/entry/K05131) | interferon alpha and beta receptor subunit 2 |
| [IFNGR1](https://www.genome.jp/entry/K05132) | interferon gamma receptor 1 |
| [IFNGR2](https://www.genome.jp/entry/K05133) | interferon gamma receptor 2 |
| JAK1 | Janus kinase 1 |
| JAK2 | Janus kinase 2 |
| JAK3 | Janus kinase 3 |
| TYK2 | tyrosine kinase 2 |
| [STAT1](https://www.genome.jp/entry/K11220) | signal transducer and activator of transcription 1 |
| [STAT2](https://www.genome.jp/entry/K11221) | signal transducer and activator of transcription 2 |
| [STAT3](https://www.genome.jp/entry/K04692) | signal transducer and activator of transcription 3 |
| [STAT4](https://www.genome.jp/entry/K11222) | signal transducer and activator of transcription 4 |
| [STAT5A](https://www.genome.jp/entry/K11223) | signal transducer and activator of transcription 5A |
| [STAT5B](https://www.genome.jp/entry/K11224) | signal transducer and activator of transcription 5B |
| [STAT6](https://www.genome.jp/entry/K11225) | signal transducer and activator of transcription 6 |
| [IRF9](https://www.genome.jp/entry/K04693) | interferon regulatory factor 9 |
| EIF2AK2 | eukaryotic translation initiation factor 2 alpha kinase 2 |
| [TLR4](https://www.genome.jp/entry/K10160) | toll like receptor 4 |
| [TICAM2](https://www.genome.jp/entry/K05409) | toll like receptor adaptor molecule 2 |
| [TICAM1](https://www.genome.jp/entry/K05842) | toll like receptor adaptor molecule 1 |
| [TLR3](https://www.genome.jp/entry/K05401) | toll like receptor 3 |
| [ZBP1](https://www.genome.jp/entry/K12965) | Z-DNA binding protein 1 |
| USP21 | ubiquitin specific peptidase 21 |
| [SQSTM1](https://www.genome.jp/entry/K14381) | sequestosome 1 |
| [HSP90AA1](https://www.genome.jp/entry/K04079) | heat shock protein 90 alpha family class A member 1 |
| [HSP90AB1](https://www.genome.jp/entry/K04079) | heat shock protein 90 alpha family class B member 1 |
| TNFAIP3 | TNF alpha induced protein 3 |
| PARP1 | poly(ADP-ribose) polymerase 1 |
| [BID](https://www.genome.jp/entry/K04726) | BH3 interacting domain death agonist |
| [BAX](https://www.genome.jp/entry/K02159) | BCL2 associated X, apoptosis regulator |
| [AIFM1](https://www.genome.jp/entry/K04727) | apoptosis inducing factor mitochondria associated 1 |
| H2AFX | H2A histone family, member X |
| HIST2H2AC | histone cluster 1 H2A family member C |
| HIST1H2AH | histone cluster 1 H2A family member H |
| HIST1H2AA | histone cluster 1 H2A family member A |
| HIST3H2A | histone cluster 3 H2A family member A |
| H2AFB3 | [H2A histone family, member B3](https://www.ncbi.nlm.nih.gov/gene/703044) |
| HIST1H2AE | histone cluster 1 H2A family member E |
| HIST1H2AB | histone cluster 1 H2A family member B |
| H2AFY2 | [H2A histone family, member Y2](https://www.ncbi.nlm.nih.gov/gene/703044) |
| H2AFY | [H2A histone family, member Y](https://www.ncbi.nlm.nih.gov/gene/703044) |
| HIST2H2AA4 | histone cluster 2 H2A family member A4 |
| H2AFJ | [H2A histone family, member J](https://www.ncbi.nlm.nih.gov/gene/703044) |
| H2AFB1 | [H2A histone family, member B1](https://www.ncbi.nlm.nih.gov/gene/703044) |
| HIST1H2AM | histone cluster 1 H2A family member M |
| HIST2H2AA3 | histone cluster 2 H2A family member A3 |
| HIST1H2AG | histone cluster 1 H2A family member G |
| HIST2H2AB | histone cluster 2 H2A family member B |
| H2AFV | [H2A histone family, member V](https://www.ncbi.nlm.nih.gov/gene/703044) |
| HIST1H2AD | histone cluster 1 H2A family member D |
| H2AFZ | [H2A histone family, member Z](https://www.ncbi.nlm.nih.gov/gene/703044) |
| HIST1H2AK | histone cluster 1 H2A family member K |
| HIST1H2AC | histone cluster 1 H2A family member C |
| HIST1H2AI | histone cluster 1 H2A family member I |
| HIST1H2AJ | histone cluster 1 H2A family member J |
| HIST1H2AL | histone cluster 1 H2A family member L |
| H2AFB2 | [H2A histone family, member B2](https://www.ncbi.nlm.nih.gov/gene/703044) |
| PPIA | peptidylprolyl isomerase A |
| [BCL2](https://www.genome.jp/entry/K02161) | BCL2 apoptosis regulator |
