# Supplementary material for: Pan-cancer analysis of necroptosis-related gene signature for the identification of prognosis and immune significance
Source: Discov Oncol. 2022 Mar 21;13:17. doi: 10.1007/s12672-022-00477-2 (PMC8938586; doi:10.1007/s12672-022-00477-2)
Supplement: Supplementary file 2 — (DOCX 16 KB) [file 12672_2022_477_MOESM2_ESM.docx]

**Risk score calculation formulas:**

**ACC:**

Risk score = 0.230298932465898 × TRAF2 + 0.342738335266177 × HMGB1 - 0.0274604685889637 × JAK3 + 0.464532471355915 × IRF9 - 0.100804518606649 × HSP90AB1 + 0.370056210284992 × PPIA + 0.994301391319621 × H2AFX + 0.251653013080674 × H2AFY

**CESC:**

Risk score = 0.142606961517936 × TNF - 0.286441021110195 × SLC25A5 + 0.188957165634617 × VDAC1 + 0.117271017121659 × FTH1 + 0.233692723981076 × IL1B + 0.634781325290046 × CHMP4C - 0.385256915489608 × BCL2 - 0.216191581281094 × HIST1H2AE - 0.26199604313502 × HIST1H2AI

**LAML:**

Risk score = 0.254398577865413 × FADD + 0.0418777322578159 × FTH1 + 0.307407568939326 × PLA2G4A + 0.0814626253503179 × AIFM1

**LGG:**

Risk score = 0.333565905753237 × CFLAR-0.210860732041259 × GLUD1 + 0.0326362004153184 × PLA2G4A + 0.126466718943175 × STAT1 + 0.00485995393057329 × EIF2AK2 + 0.339271539688111 × H2AFX - 0.0705207515549953 × HIST3H2A - 0.230813018648307 × H2AFY2

**LIHC:**

Risk score = 0.0297780473746593 × PYGB - 0.101113274662343 × IL33 + 0.239918552200294 × USP21 + 0.238099040037305 × SQSTM1 + 0.201184104906853 × PPIA + 0.0801568770331508 × H2AFX + 0.0981490076561848 × H2AFZ

**PAAD:**

Risk score = 0.0937624798908577 × BIRC3 + 0.162163203925034 × PYGL + 0.144856427655683 × PYGB + 0.344806436998224 × TNFSF10 + 0.0914925687412144 × TNFRSF10A - 0.551636257836531 × TYK2 + 0.000686874561700975 × STAT1 + 0.0728986549213852 × HIST1H2AC

**SKCM:**

Risk score = 0.200898376329362 × VDAC1 + 0.120382358200087 × PLA2G4E + 0.226073291365733 × PLA2G4D + 0.0569799964318873 × PGAM5 + 0.0368198716641599 × CHMP4C-0.0198860299379785 × FASLG-0.128642604153774 × IFNGR2 - 0.208501959753085 × STAT1-0.109759990971266 × IRF9 + 0.252360119262102 × PARP1 + 0.0899969943909046 × H2AFJ + 0.0558844539653719 × H2AFZ

**THYM:**

Risk score = 0.342688972678846 × TNF + 0.176030071347679 × SLC25A4 + 0.527739773662039 × CASP1 + 0.28610268987457 × TNFSF10 - 0.573984660278325 × IFNAR2 + 0.218371189189496 × IFNGR2 + 0.267097780436071 × JAK2+0.176687995773885 × STAT4 + 0.307030607723113 × H2AFY2
