# Supplementary figures and images for: Pan-cancer analysis of necroptosis-related gene signature for the identification of prognosis and immune significance
Source: Discov Oncol. 2022 Mar 21;13:17. doi: 10.1007/s12672-022-00477-2 (PMC8938586; doi:10.1007/s12672-022-00477-2)

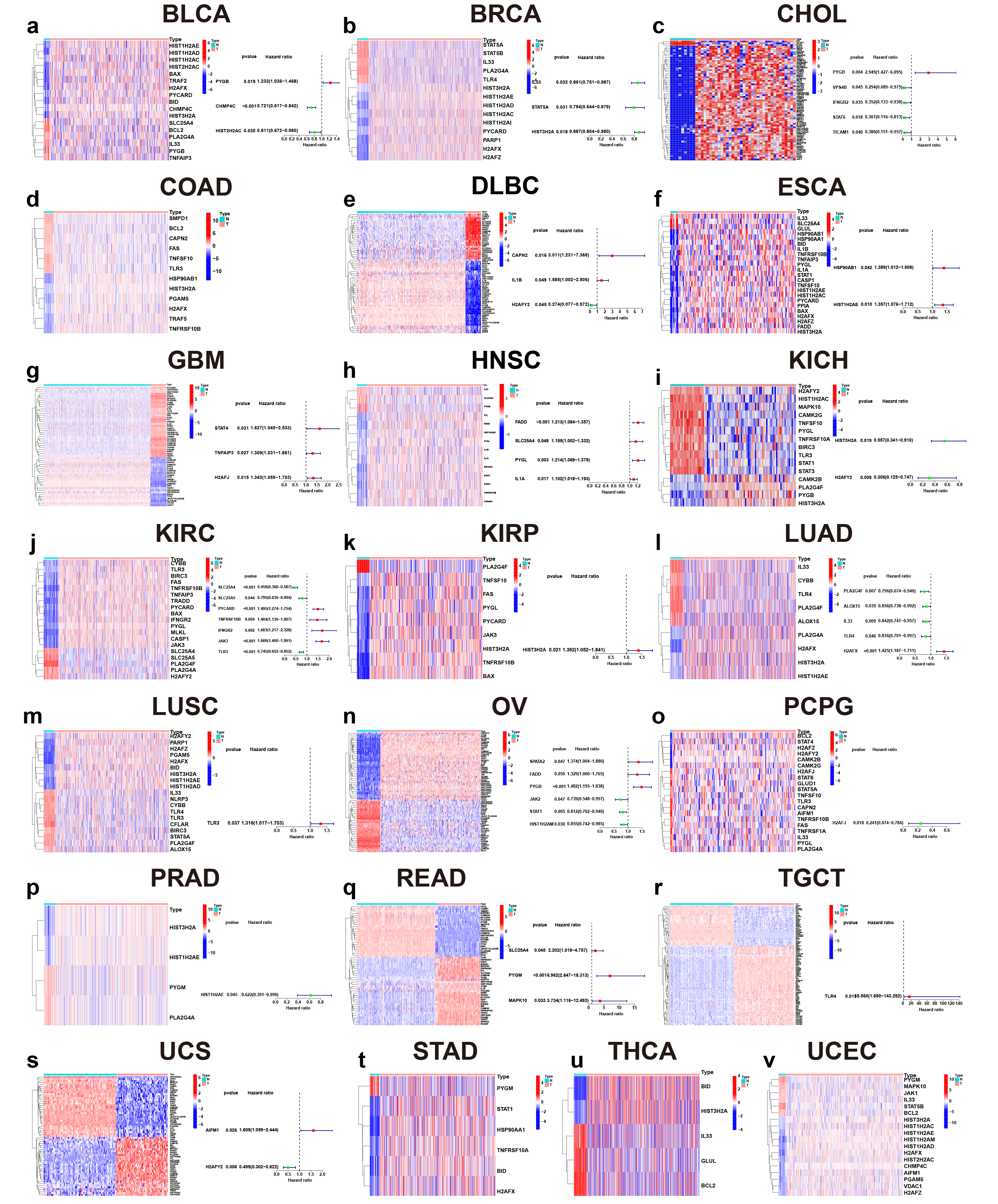

Supplement: Supplementary file 3 — Figure S1. Differentially expressed necroptosis-related genes (DENGs) and survival analysis in other cancers. The heat maps and forest plots showed the expression state and the prognostic effect of the DENGs in bladder urothelial carcinoma (BLCA) (a), breast invasive carcinoma (BRCA) (b), cholangiocarcinoma (CHOL) (c), colon adenocarcinoma (COAD) (d), lymphoid neoplasm diffuse large B-cell lymphoma (DLBC) (e), esophageal carcinoma (ESCA) (f), glioblastoma multiforme (GBM) (g), head and neck squamous cell carcinoma (HNSC) (h), kidney chromophobe (KICH) (i), kidney renal clear cell carcinoma (KIRC) (j), kidney renal papillary cell carcinoma (KIRP) (k), lung adenocarcinoma (LUAD) (l), lung squamous cell carcinoma (LUSC) (m), ovarian serous cystadenocarcinoma (OV) (n), pheochromocytoma and paraganglioma (PCPG) (o), prostate adenocarcinoma (PRAD) (p), rectum adenocarcinoma (READ) (q), testicular germ cell tumors (TGCT) (r), uterine carcinosarcoma (UCS) (s), stomach adenocarcinoma (STAD) (t), thyroid carcinoma (THCA) (u), uterine corpus endometrial carcinoma (UCEC) (v). |log2 (fold change)| > 1 and false discovery rate (FDR) < 0.05 were used as the screening criteria for the detection of DENGs between tumor and normal tissues. Logrank p value and hazard ratio were presented beside each forest plot (TIF 9868 KB) [file 12672_2022_477_MOESM3_ESM.tif]

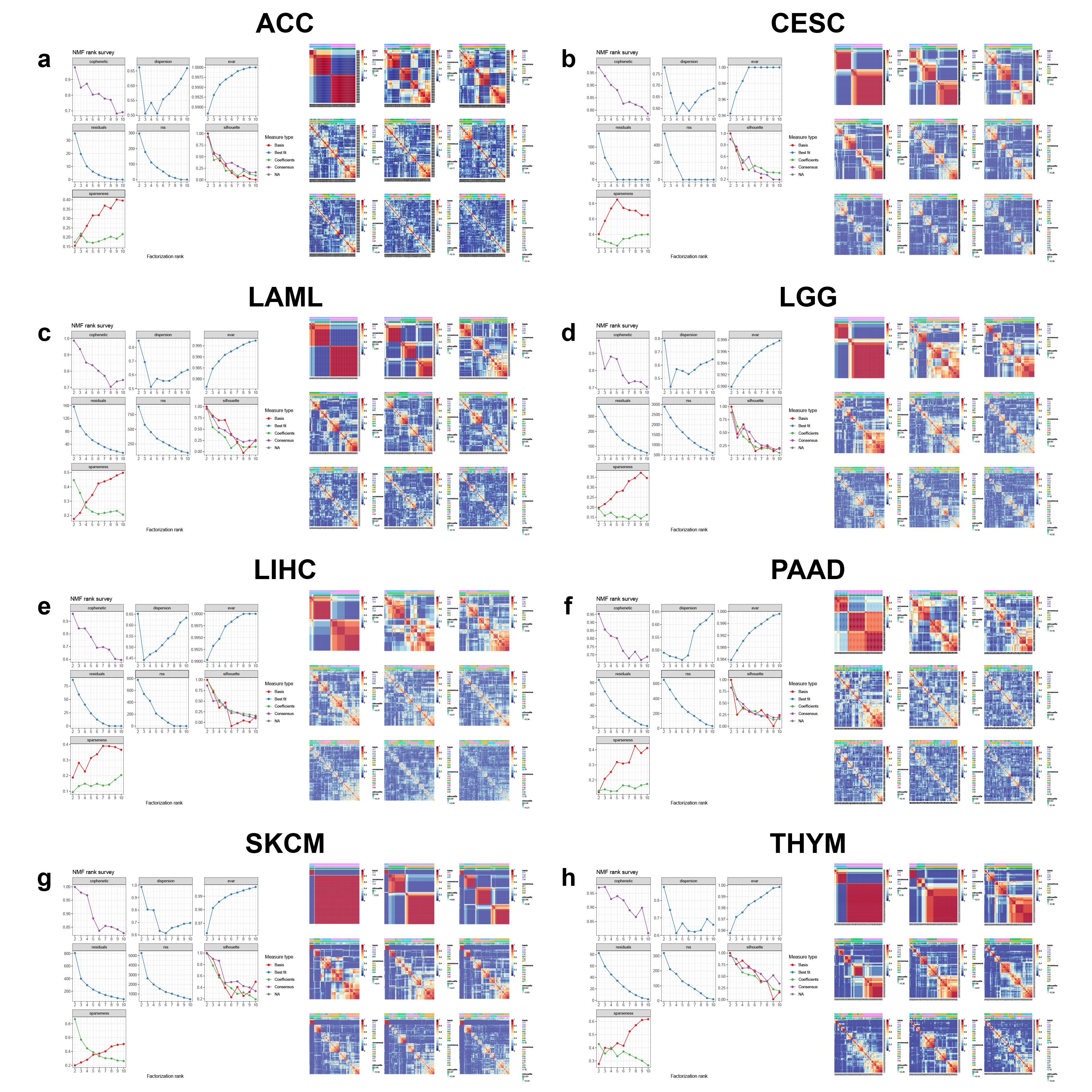

Supplement: Supplementary file 4 — Supplementary 4 Figure S2. Comprehensive correlation coefficient and the consensus matrix heat maps of NMF analysis. The NMF rank survey and the consensus matrix heat maps were exhibited with K values ranking from 2 to 10 in adrenocortical carcinoma (ACC) (a), cervical squamous cell carcinoma endocervical adenocarcinoma (CESC) (b), acute myeloid leukemia (LAML) (c), brain lower grade glioma (LGG) (d), liver hepatocellular carcinoma (LIHC) (e), pancreatic adenocarcinoma (PAAD) (f), skin cutaneous melanoma (SKCM) (g) and thymoma (THYM) (h) (TIF 8507 KB) [file 12672_2022_477_MOESM4_ESM.tif]
